# Identification of intracellular bacteria from multiple single-cell RNA-seq platforms using CSI-Microbes

**DOI:** 10.1126/sciadv.adj7402

**Published:** 2024-07-03

**Authors:** Welles Robinson, Joshua K. Stone, Fiorella Schischlik, Billel Gasmi, Michael C. Kelly, Charlie Seibert, Kimia Dadkhah, E. Michael Gertz, Joo Sang Lee, Kaiyuan Zhu, Lichun Ma, Xin Wei Wang, S. Cenk Sahinalp, Rob Patro, Mark D. M. Leiserson, Curtis C. Harris, Alejandro A. Schäffer, Eytan Ruppin

**Affiliations:** ^1^ Cancer Data Science Laboratory, Center for Cancer Research, National Cancer Institute, Bethesda, MD 20892, USA.; ^2^ Center for Bioinformatics and Computational Biology, University of Maryland, College Park, MD 20910, USA.; ^3^ Department of Computer Science, University of Maryland, College Park, MD 20910, USA.; ^4^ Surgery Branch, Center for Cancer Research, National Cancer Institute, Bethesda, MD 20892, USA.; ^5^ Tumour Immunogenomics and Immunosurveillance Laboratory, Department of Oncology, University College London, London, UK.; ^6^ Laboratory of Human Carcinogenesis, Center for Cancer Research, National Cancer Institute, Bethesda, MD 20892, USA.; ^7^ Center for Cancer Research Single Cell Analysis Facility, Frederick National Laboratory for Cancer Research, Bethesda, MD 20701, USA.; ^8^ Department of Artificial Intelligence and Department of Precision Medicine, School of Medicine, Sungkyunkwan University, Suwon 16419, Republic of Korea.; ^9^ Department of Computer Science, Indiana University, Bloomington, IN 47408, USA.; ^10^ Department of Computer Science and Engineering, University of California, San Diego, La Jolla, CA 92093, USA.

## Abstract

The study of the tumor microbiome has been garnering increased attention. We developed a computational pipeline (CSI-Microbes) for identifying microbial reads from single-cell RNA sequencing (scRNA-seq) data and for analyzing differential abundance of taxa. Using a series of controlled experiments and analyses, we performed the first systematic evaluation of the efficacy of recovering microbial unique molecular identifiers by multiple scRNA-seq technologies, which identified the newer 10x chemistries (3′ v3 and 5′) as the best suited approach. We analyzed patient esophageal and colorectal carcinomas and found that reads from distinct genera tend to co-occur in the same host cells, testifying to possible intracellular polymicrobial interactions. Microbial reads are disproportionately abundant within myeloid cells that up-regulate proinflammatory cytokines like *IL1*Β and *CXCL8*, while infected tumor cells up-regulate antigen processing and presentation pathways. These results show that myeloid cells with bacteria engulfed are a major source of bacterial RNA within the tumor microenvironment (TME) and may inflame the TME and influence immunotherapy response.

## Teaser

The capture rate of reads from intracellular bacteria versus contaminants depends on the scRNA-seq technology and chemistry.

## INTRODUCTION

In addition to malignant and nonmalignant human cells, the tumor microenvironment (TME) consists of microbes including viruses, bacteria, and fungi, collectively referred to as the tumor microbiome. Early studies of the tumor microbiome focused on viruses that are estimated to cause ~10 to 15% of human cancers worldwide, including Merkel cell polyomavirus, which is detectable in ~75% of Merkel cell carcinomas and Hepatitis B and C viruses, which are collectively estimated to cause more than 60% of liver cancers (*1*–*4*).

Some more recent experimental and computational studies expanded the scope of the tumor microbiome to include tumor-resident bacteria and fungi (*5*–*8*). For example, early studies of bacteria in tumors reported the increased prevalence of the bacterium *Fusobacterium nucleatum* in colorectal carcinoma compared to adjacent nontumor tissue (*9*, *10*). Larger-scale reports demonstrate that many, if not all, solid tumor types have a microbiome, possibly distinct and distinguishable from the microbiome of nearby nontumor tissue (*6*, *8*–*10*). Further studies have demonstrated the functional importance of specific members of the tumor microbiome to multiple hallmarks of tumorigenesis including mutagenesis, metastasis, and immune evasion as well as response to chemotherapy (*9*–*15*). Tumor microbiome studies have shifted partly to intracellular microbes due to recent findings that the vast majority of intratumoral bacterial taxa, including *F. nucleatum*, appear to reside intracellularly within the TME (*6*, *7*, *16*). Despite these advances, it has remained an important, open challenge to identify which microbial taxa reside intracellularly and whether they reside exclusively or preferentially inside tumor cells, immune cells, or cells of the noncancerous tissue adjacent to the solid tumor.

One increasingly popular approach for studying intracellular or host cell–associated microbes is the analysis of microbial reads from single-cell RNA sequencing (scRNA-seq) in the settings of viral infection or of cancer (*17*–*21*). To provide context for our work, we mention two recently published reports analyzing microbial reads from scRNA-seq in the context of cancer (*19*, *21*). One of these studies introduced a computational approach (SAHMI) for decontaminating droplet-based scRNA-seq data, which it applied to analyze two 10x 3’ v2 datasets of pancreatic cancer (*19*). The other introduced an experimental scRNA-seq approach and analysis pipeline termed INVADE-seq that aims to increase the capture of bacterial reads by including a primer for a conserved region of the bacterial 16*S* ribosomal RNA (rRNA) in 10x 5′ polyA capture, which was applied to analyze seven oral squamous cell carcinomas (*21*). These studies present distinct methods for filtering out potential environmental contaminants (*22*), which have been a concern with the application of traditional 16*S* rRNA-seq and bulk DNA- and RNA-seq–based microbiome studies. SAHMI uses multiple decontamination steps, while the INVADE-seq study does not discuss methods for filtering potential contaminants. While making important contributions, each of these two studies focused on studying one single scRNA-seq wet laboratory method, leaving open the challenge of providing comparative guidance regarding scRNA-seq technology selection for future scRNA-seq studies that profile the microbiome.

**Fig. 1.**
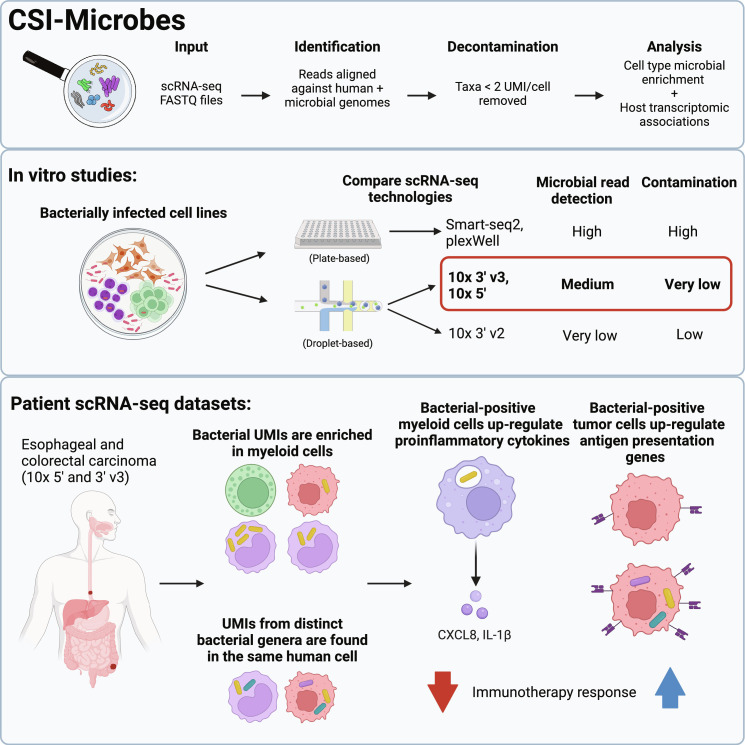
Overview of CSI-Microbes. Overview of the CSI-Microbes analysis pipeline for analysis of intracellular bacteria from scRNA-seq datasets (a more detailed overview is available in fig. S1A). Key findings of the differences in detection of microbial reads between scRNA-seq technologies from analysis of human cells infected in vitro from both this study and previous studies. Results from CSI-Microbes applied to scRNA-seq of patient esophageal and colorectal carcinoma including enrichment of bacterial UMIs in myeloid cells, co-occurrence of bacterial UMIs in the same human cells, and myeloid and tumor cell–specific transcriptomic associations with bacterial UMIs.

To compare microbial read detection of different scRNA-seq technologies, we developed a reproducible computational pipeline, named CSI-Microbes. We then comprehensively applied CSI-Microbes to study multiple plate-based (Smart-seq2 and plex-Well) and droplet-based (10x 3’ v2, 3’ v3, and 5’) scRNA-seq datasets of human cells exposed in vitro to select bacteria. Specifically, we found that plate-based technologies capture the most microbial reads, but approximately half of these microbial reads map to putative contaminant genera (Fig. 1). By comparison, 10x technologies capture relatively few reads from putative contaminants, but more successfully capture reads mapping to the in vitro–exposed bacteria (Fig. 1). Their capture levels depend on the specific chemistry, with at least an order of magnitude more microbial reads detected by the newer 10x chemistries (10x 3’ v3 and 10x 5’) compared to the earlier 10x 3’ v2 chemistry. These findings thus identify the newer 10x protocols as the preferred methods for studying the tumor microbiome (Fig. 1).

Armed with these insights, we next applied CSI-Microbes to interrogate the intratumoral microbiome of patient colorectal and esophageal carcinomas by analyzing large, recently published 10x 3’ v3 and 5’ datasets. We show that intracellular bacteria are disproportionately found in myeloid cells and have identified bacterial genera that co-occur in the same cells more than expected under a random model (Fig. 1). Last, we combined the microbial and host cell transcriptomic reads to reveal cell type–specific transcriptomic signatures that are shared between in vitro–and in vivo–infected cells (Fig. 1). Those include the up-regulation of antigen processing and presentation pathways in infected tumor cells and the up-regulation of proinflammatory cytokines *IL1Β* and *CXCL8* in myeloid cells with bacteria engulfed, whose potential therapeutic relevance is discussed.

Our work makes four contributions. First and foremost, we developed a new pipeline, CSI-Microbes, for identifying microbial reads from both plate- and droplet-based scRNA-seq technologies, distinguishing intracellular bacteria reads from those of contaminants and determining cell types enriched with bacterial reads. The second most important is that we produced a new dataset, herein called Robinson2023, of multiple cell lines exposed in vitro to *F. nucleatum* with appropriate controls sequenced with multiple scRNA-seq technologies. Leveraging our first two contributions, we compared capture rates for intracellular bacteria and contaminants across different scRNA-seq technologies and found that 10x 3’ v3 and 5’ chemistries are superior to earlier chemistries and plate-based approaches. Last, we applied CSI-Microbes to colorectal and esophageal patient 10x 3’ v3 and 5’ datasets to find an enrichment of bacterial reads in myeloid cells within the TME.

## RESULTS

### Overview of CSI-Microbes

We developed a computational pipeline, CSI-Microbes (https://github.com/ruppinlab/CSI-Microbes-identification), to identify microbial reads from plate-based and 10x scRNA-seq datasets [when comparing plate-based and 10x datasets, we use the word “reads” to refer to unique molecular identifiers (UMIs) when describing 10x data for consistency] (fig. S1A). The modules of CSI-Microbes are described in Materials and Methods; for some of the more technical parts of Results, it is useful to be aware that the module that aligns reads to genomes can use different approaches for aligning nonhuman reads to microbial genome(s) including PathSeq (*23*) (which was used also by the INVADE-seq study) and CAMMiQ (*24*) to align to many microbial genomes and SRPRISM (*25*) to align to a (few) specific microbial genome(s). Unless otherwise specified, the results presented in this study use the PathSeq option for sequence alignment. In total, we applied CSI-Microbes to five different datasets (Table 1).

**Table 1.** Datasets analyzed.

**Abbreviation**	**scRNA-seq technology**	**Description**	**NCBI Bioproject Accession**	**Source/reference**
Aulicino2018	Smart-seq2 (plate-based)	moDCs exposed in vitro to *S. enterica*	PRJNA437328	(*27*)
Ben-Moshe2019	10x 3’ v2	PBMCs exposed in vitro to *S. enterica*	PRJNA503437	(*28*)
Robinson2023	plexWell (plate-based) and 10x 3’ v3 and 5’ v2	HCT116, THP1 and Jurkat T exposed in vitro to *F. nucleatum*	PRJNA970826	This paper
Pelka2021	10x 3’ v2 and 3’ v3	Colorectal carcinoma	PRJNA723926	(*29*)
Zhang2021	10x 5’	Esophageal carcinoma	PRJNA672851	(*33*)

Results is composed of two main parts, A and B, each consisting of a few subsections. Part A describes a series of controlled experiments and analyses that evaluate the efficacy of different scRNA-seq technologies in uncovering the microbiome of infected cells in vitro. In part B, we analyze patients’ tumor data using the most informative sequencing platforms identified in part A to interrogate host-microbe interactions.

### Evaluating CSI-Microbes across sequencing platforms

#### CSI-Microbes identifies reads from known intracellular microbes from specifically designed plate- and droplet-based scRNA-seq technologies

**Fig. 2.**
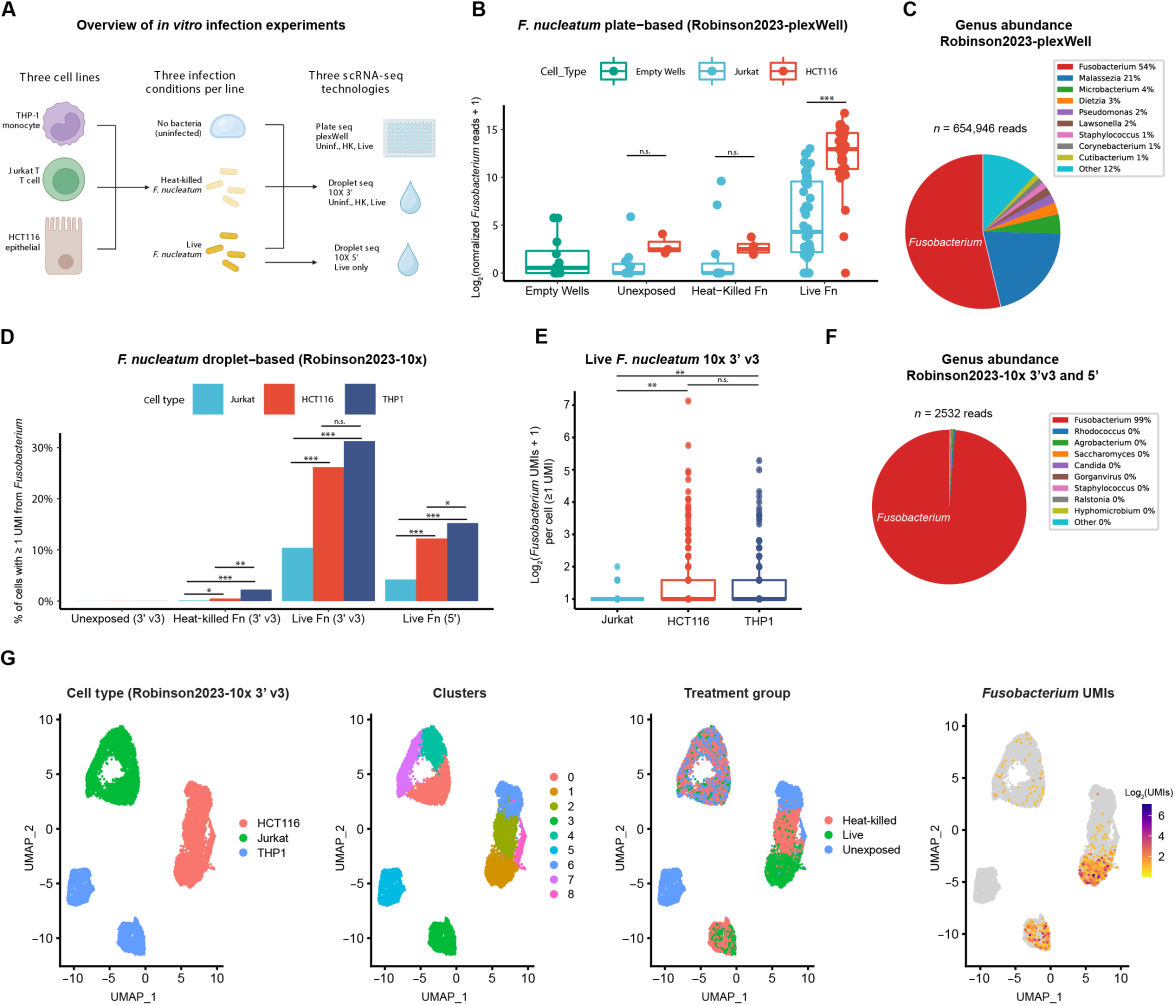
Comparison of the performance of CSI-Microbes on droplet versus plate-seq platforms. (**A**) Experimental design for *F. nucleatum* (*Fn*) exposure and scRNA-seq for this paper (Robinson2023). (**B**) The number of reads (spike-in normalized and log_2_ transformed) per cell mapping to the genus *Fusobacterium* from Jurkat T and HCT116 cells grouped by cell type and exposure condition and sequenced using plate-based scRNA-seq (Robinson2023-plexWell). (**C**) The percentage of genera-resolution microbial reads mapped to *Fusobacterium* and other genera (suspected contaminants) from live-*Fn*–exposed cells sequenced using plexWell. (**D**) The percentage of cells with at least one read from *Fusobacterium* from Jurkat T, HCT116, and THP1 cells grouped by cell type and exposure condition sequenced using 10x 3’ v3 and 5’ (Robinson2023). We chose to use bar plots because most of the data points have the value 0. (**E**) The number of UMIs from genus *Fusobacterium* per cell grouped by cell line (only cells with ≥1 *Fusobacterium* UMI were included) (**F**) The percentage of genera-resolution microbial reads mapped to *Fusobacterium* and other genera (suspected contaminants) from live-*Fn*–exposed cells sequenced using 10x 3’ v3 and 5’. (**G**) UMAP visualization of cells sequenced using 10x 3’ v3 including cell type, transcriptomic cluster, treatment group, and *Fusobacterium* UMIs. *P < 0.05; **P < 0.01; ***P < 0.001. For statistics used to determine *P* values, see Cell type enrichment (plate-based protocols) and Cell type enrichment (10x Genomics protocols) subsections of Materials and Methods. n.s., not significant.

To compare the capture of bacterial reads by different scRNA-seq technologies, we designed in vitro infection experiments, which we assayed using multiple scRNA-seq technologies (Fig. 2A). First, we selected three scRNA-seq technologies to compare using the same experimental design: 10x 3’ v3 and 10x 5’ (droplet) and plexWell (plate). Second, we included three cell lines of various tissues of origin, including epithelial cells (HCT116, colorectal cancer), monocytes (THP1), and T cells (Jurkat T). Third, we used three experimental treatment groups for each cell line: naïve or unexposed cells for background microbial UMI signal, heat-killed *F. nucleatum* (HK-*Fn* exposed), and live *F. nucleatum*–exposed cells (live-*Fn* exposed). Fourth, we included empty wells in the plexWell capture to account for well carryover as a possible source of contamination.

We evaluated the invasion by *F. nucleatum* of the three cell lines using two orthogonal approaches. First, using an RNAscope (*26*) probe specific for *F. nucleatum* 23*S* rRNA, we observed that 25, 70, and 80% of live-*Fn*–exposed Jurkat T, THP1, and HCT116 cells were positive for *F. nucleatum* (fig. S1B). Second, we measured colony-forming units (CFUs) following cell lysis to compare the number of live intracellular bacteria and only recovered viable bacteria from live-*Fn*–exposed HCT116 cells (fig. S1C). Combining the results from the two approaches suggests that while the detection of bacterial RNA does not imply the presence of live intracellular bacteria, higher levels of bacterial RNA are associated with live intracellular bacteria, except for THP1, which we discuss later.

From the plate-based dataset, we identified 393,380 *Fusobacterium* reads (mean, 4574 per cell) from live-*Fn*–exposed cells, including significantly more *Fusobacterium* reads in HCT116 cells compared to Jurkat T cells and 202 reads (mean, 13 reads per cell) from HK-*Fn*–exposed cells (Fig. 2B and fig. S2 and S3). Unexpectedly, we identified 292 *Fusobacterium* reads from unexposed cells and empty wells (mean, 11 reads per cell) likely from cross-well contamination (Fig. 2B). We also found many reads from putative contaminants (other, unexpected bacterial, fungal, and viral genera), which comprised 45% (317,250 of 710,630) of the genera-resolution microbial reads from live-*Fn*–exposed cells (Fig. 2C and table S1). The large number of reads assigned by PathSeq to a non-*Fusobacterium* genus could indicate either contamination or some algorithmic flaw in PathSeq due to its heuristic alignment method and incomplete genome database. To investigate the latter possibility, we identified all 1,223,238 reads assigned by PathSeq to a taxon that is at genus level or below and does not belong to genus *Fusobacterium*. Among these reads, 4889 (0.4%) had a significant alignment to *Fusobacterium*, which we defined formally as a blastn match of *E* value <1 × 10^−15^ to at least 1 genome among 16 *Fusobacterium* genomes (table S1D), which include all those used by PathSeq and more; in this analysis, blastn was run with word size 11, which is the lowest value allowed and hence finds the most possible matches. Among these 4889 reads, a small proportion (~5%) were assigned by PathSeq to a different genus in order *Fusobacteriales* due to incomplete coverage in PathSeq’s database, but the vast majority were completely mis-assigned, indicating that they come from sequences that are similar in highly divergent bacteria. We conclude that PathSeq has a modest (0.4%) false positive rate, but the vast majority of sequences classified in table S1A as non-*Fusobacterium* are correctly classified as something other than *Fusobacterium* and got into the samples by wet laboratory contamination.

From the droplet-based datasets, we identified 1401 and 926 *Fusobacterium* UMIs from live-*Fn*–exposed cells sequenced using 10x 3’ v3 (mean 0.51 UMIs per cell) and 10x 5’ (mean, 0.19 UMIs per cell), respectively (fig. S4), and this difference was significant after correcting for sequencing depth (8.1 versus 3.1 *Fusobacterium* UMIs per million human reads for 3′ v3 and 5′, respectively; *P* < 2.2 × 10^−16^). We observed that most *Fusobacterium-*positive (≥1 UMI) cells were HCT116 and THP1 (Fig. 2D), and *Fusobacterium-*positive HCT116 and THP1 cells had more *Fusobacterium* UMIs per cell compared to Jurkat T (Fig. 2E). We identified 21 *Fusobacterium* UMIs from HK-*Fn*–exposed cells (67% from THP1 cells) and 0 from unexposed cells. We found relatively few UMIs from putative contaminants, which made up 2% (21 of 947) and 1% (10 of 1411) of the microbial reads from live-*Fn*–exposed cells sequenced using 10x 5′ and 3′ v3, respectively (Fig. 2F and table S1).

Next, we examined the microbial UMIs associated with empty droplet cell barcodes in samples exposed to live *F. nucleatum* and found more microbial UMIs from empty-droplet barcodes compared to cell-associated barcodes (10,676 versus 2532) of which 98% (10,462 of 10,676) microbial UMIs mapped to *Fusobacterium* (fig. S4). These results suggest that empty droplets, which (unlike empty wells) contain low-quality cells and are filtered out, can be retained and used for microbial read detection at the sample level but not at the single-cell level (as done by SAHMI). Furthermore, empty droplets should not be used for identification or filtering of contaminant genera.

Next, we overlayed *Fusobacterium* reads and treatment groups onto cell clusters derived using only human genes, which revealed that host transcriptomic changes to bacteria were cell line dependent (Fig. 2G and fig. S1D). The host transcriptomic clusters of Jurkat T cells, which were the least infected cell line, did not separate by treatment groups. In contrast, the clusters of HCT116 cells, which were highly infected, showed clear treatment group-specific subclustering. While one cluster of THP1 cells included almost all unexposed cells, the other THP1 cluster contained a mix of live-*Fn*–exposed and HK-*Fn*–exposed cells. The similar response to live and heat-killed *F. nucleatum* suggests that THP1 cells may be engulfing the bacteria, which would explain why viable bacteria could not be cultured from lysed cells despite high levels of *Fusobacterium* RNA detected with RNAscope (Materials and Methods) and scRNA-seq.

#### CSI-Microbes and INVADE-seq provide improved sensitivity in microbial read identification compared to SAHMI

**Fig. 3.**
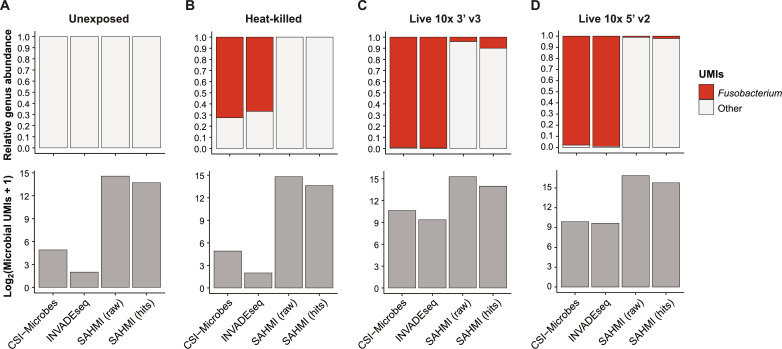
CSI-Microbes, INVADEseq, and SAHMI comparison using Robinson2023-10x. (**A**) Each bar represents average relative (top) or absolute (bottom) abundance for all cell lines not exposed to *F. nucleatum* within Robinson2023-10x, as calculated by each package. SAHMI (raw) and (hits) refer to the total microbial UMIs identified and those which passed the contamination filtering, respectively. (**B** to **D**) As described in (A), separated by *F. nucleatum* treatment group: heat-killed bacteria (B), live bacteria sequenced with 3’ v3 (C) or 5’ v2 (D).

Next, we compared CSI-Microbes to the SAHMI and INVADE-seq pipelines on the Robinson2023-10x dataset (Materials and Methods). We did not compare the tools on Robinson2023-plexWell because INVADE-seq explicitly only supports 10x datasets, while SAHMI implicitly assumes the presence of cell barcodes and UMIs, which are not used in many plate-based protocols, including those used to generate Robinson2023-plexWell and Aulicino2018. We report two sets of results for the SAHMI comparison: SAHMI-raw, which includes all microbial reads, and SAHMI-hits, which includes only reads from genera that pass the SAHMI decontamination tests. SAHMI reported two genera to be “hits” from each of the 3′ v3 samples (unexposed, heat-killed, and live-exposed) and 32 genera to be hits from the 5′ v2 live-exposed sample. SAHMI identified *Fusobacterium* to be a hit in both the 3′ v3 and 5′ live-exposed samples. Across all four samples, SAHMI-raw and SAHMI-hits identified more microbial reads than CSI-Microbes and INVADE-seq (211,605 and 97,949 versus 2590 and 1462, respectively) (Fig. 3). However, a much smaller percentage of microbial reads identified by SAHMI-raw and SAHMI-hits mapped to *Fusobacterium* compared to CSI-Microbes and INVADE-seq (1.8 and 3.9% versus 98.7 and 99.3%, respectively). Thus, we conclude that CSI-Microbes performs much more accurately than SAHMI on Robinson2023-10x. By varying some parameter settings, we further determined that nearly all the differences between CSI-Microbes and INVADE-seq could be explained by more permissive PathSeq parameters used by CSI-Microbes, especially the alignment length required (36 bases in CSI-Microbes versus 60 bases in INVADE-Seq). The simplest to state advantage of CSI-Microbes over INVADE-Seq is that CSI-Microbes can analyze all available scRNA-seq data types, while INVADE-Seq is limited to only 10x datasets. Another important advantage of CSI-Microbes is the set of downstream analysis tools for enrichment, which has no counterpart in INVADE-Seq.

To evaluate whether our findings could be generalized to other datasets, we then analyzed two publicly available scRNA-seq datasets of human cells exposed in vitro to the known invasive bacterium *Salmonella enterica* in a controlled manner. The first dataset, which we term Aulicino2018 (Table 1), is a plate-based scRNA-seq (Smart-seq2) dataset of monocyte-derived dendritic cells (moDCs) exposed to *S. enterica* (live-*Se* exposed) as well as unexposed control cells (*27*). The authors divided the live-*Se*–exposed moDCs into “infected” or “bystander” cells depending on whether the presence of CellTrace-labeled *S. enterica* could be detected using fluorescence-activated cell sorting (FACS). Like our analysis of Robinson2023-plexWell, we identified 756,327 reads (mean, 2886 reads per cell) mapping to the *Salmonella* genus, which were enriched in the infected cells but still detected in unexposed control cells (fig. S5A). As in Robinson2023-plexWell, we detected high levels of putative contaminants as 57% (986,581 of 1,742,908) of the microbial reads in live-*Se*–exposed moDCs mapped to genera other than *Salmonella* (fig. S5B and table S1).

The second dataset, which we term Ben-Moshe2019 (Table 1), is a droplet-based (10x 3’ v2) scRNA-seq dataset of human peripheral blood mononuclear cells (PBMCs) exposed to *S. enterica* as well as unexposed control cells (*28*). FACS analysis in the original study revealed that monocytes were enriched for the red fluorescent protein expressed by *S. enterica* in the authors’ model system. In stark contrast to our results from Robinson2023-10x, we only observed eight UMIs mapping to *Salmonella* from Ben-Moshe2019 (mean, 0.0023 reads per cell), although *Salmonella*-positive cells (≥1 *Salmonella* UMI) were enriched in monocytes and *Salmonella* UMIs were not detected in the unexposed control cells (fig. S5C). We found 28 UMIs from putative contaminants, which constituted 77% (28 of 36) of the total microbial UMIs due to the low number of *Salmonella* UMIs (fig. S5D).

**Fig. 4.**
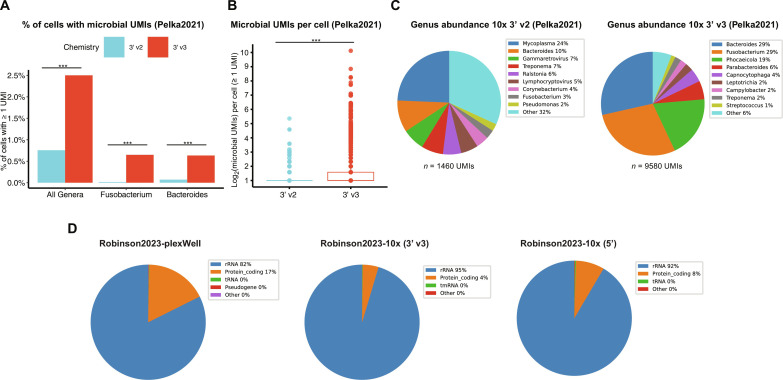
CSI-Microbes reveals differences in the number and type of bacterial RNAs captured within and between 10x chemistries and plate-based approaches. (**A**) Comparison of the percentage of cells with ≥1 microbial UMI between cells sequenced using 3’v2 and 3’ v3 by Pelka2021. We used bar plots because most of the data points have the value 0. (**B**) Comparison of the number of microbial UMIs per cell (minimum ≥1 microbial UMI) between cells sequenced using 3’ v2 and 3’ v3 by Pelka2021. (**C**) Comparison of the percentage of genera-resolution microbial reads mapped to the top 10 genera between 3’ v2 and 3’ v3 chemistries by Pelka2021. (**D**) The percentage of Fusobacterium reads belonging to the major types of RNA from Robinson2023-plex, 10x 3’ v3 and 5’. ***P < 0.001. For statistics used to determine *P* values, see Cell type enrichment (10x Genomics protocols) subsections of Materials and Methods.

Although there are important technical differences between the *F. nucleatum* and *S. enterica* invasion experiments, we hypothesized that some of the difference in number of reads captured from the infecting bacterium in the 10x datasets may be related to which chemistry was used, which has been shown to strongly influence which human genes are sequenced (https://kb.10xgenomics.com/hc/en-us/articles/360026501692-Do-we-see-a-difference-in-the-expression-profile-of-3-Single-Cell-v3-chemistry-compared-to-v2-chemistry-). To test for differences in chemistry within a single dataset, we analyzed the dataset Pelka2021 (Table 1), which is a large scRNA-seq atlas of colorectal carcinomas sequenced with either 10x 3’ v2 or 10x 3’ v3 chemistries (*29*). Although nearly twice as many cells were sequenced using 10x 3’ v2, we identified substantially more microbial reads from the tumor samples sequenced using 10x 3’ v3 compared to those sequenced using 10x 3’ v2 using the absolute number of microbial reads (9580 versus 1460 genera-resolution microbial reads, respectively), the sequencing depth–normalized number (Fisher exact test *P* < 1 × 10^−300^; odds ratio = 6.24), the percentage of cells with ≥1 microbial UMI (Fisher exact test *P* < 4.6 × 10^−260^; odds ratio = 3.4), and the number of microbial UMIs per cell (Wilcoxon rank sum test *P* = 4 × 10^−30^) (Fig. 4, A and B, and fig. S6). We also observed differences in the genera detected (Fig. 4C) and noted that a significantly higher percentage (64% or 6155 of 9580) of the 3′ v3 microbial reads map to genera found enriched in colorectal carcinoma by a meta-analysis study (*30*) compared to only 23% (342 of 1467) of the 3’ v2 microbial reads (hypergeometric enrichment test *P* < 3 × 10^−194^). The increased number of microbial UMIs combined with the higher proportion of these UMIs mapping to known resident microbes provides an estimate that 10x 3’ v3 captures 36-fold more resident microbial reads per cell compared to 10x 3’ v2.

We next aligned the nonhuman reads directly to the *F. nucleatum* genome using SRPRISM (*25*) to analyze the type of bacterial transcripts identified because bacterial mRNAs are polyadenylated at a much lower frequency and with substantially shorter polyA tails compared to eukaryotic mRNA (*31*). We found that most bacterial transcripts captured by scRNA-seq protocols are rRNA (82, 92, and 95% of plexWell, 10x 5′ and 10x 3’ v3 *F. nucleatum* reads) and not mRNA (Fig. 4D), which is reassuringly comparable to previous reports from bulk polyA-capture RNA-seq and 10x Visium spatial transcriptomics (*21*, *32*).

In summary, our analysis identified important differences between the scRNA-seq technologies and identified two important factors for the study of intracellular bacteria. The first challenge is the removal of environmental contaminants, which we demonstrate to be a bigger problem in plate-based technologies compared to droplet-based technologies, consistent with the idea that contaminants are more likely to reach an exposed well handled by people compared to droplets controlled by a microfluidics system. The second challenge is distinguishing infected cells with live, intracellular bacteria from bystander cells that may contain bacterial RNA but not live, intracellular bacteria. For example, RNA from *F. nucleatum* is detected in 6, 10, 88, and 25% of live-*Fn*–exposed Jurkat T cells using 10x 3’ v3, 10x 5’, plexWell, and RNAscope, respectively, but no live bacteria were cultured from live-*Fn*–exposed Jurkat T cells. Similarly, we detect *Salmonella* RNA from 97% (123 of 127) of bystander moDCs from Aulicino2018, although no or very few viable bacteria were recovered from bystander moDCs (a consistent number of bacteria were recovered from infected moDCs) (*27*). In both experiments, we observed significantly more bacterial reads from the cell type from which live bacteria could be cultured (except for the THP1 cells in Robinson2023).

Given the lower levels of environmental contamination from 10x compared to plate-based technologies, we focused on solving the above problems for 10x 3’ v3 and 5’. Using Robinson2023-10x, we observed that a simple threshold of ≥2 microbial UMIs per cell effectively eliminates contaminants (*Fusobacterium* was the only genus with ≥2 UMIs in a single cell) and strongly enriches for live-*Fn*–exposed HCT116 and THP1 (of the 334 cells with ≥2 *Fusobacterium* UMIs, 6 are live-*Fn*–exposed Jurkat, 1 is HK-*Fn*–exposed THP1, and the remaining 327 are live-*Fn*–exposed HCT116 and THP1) (fig. S2B). We subsequently use the term infected to refer to any cell with ≥2 UMIs from the same genus, except for myeloid-derived cells (like the THP1 cell line), which we refer to as “myeloid cells with bacteria engulfed” following (*21*) because of the difficulty distinguishing between cells with bacteria engulfed and cells with live bacteria using microbial UMIs alone. To evaluate the robustness of our key findings to the choice of UMI threshold, we repeat our key analyses of infected cells using a range of UMI cutoffs (one to five microbial UMIs).

### Interrogating the intratumoral microbiome of colorectal and esophageal carcinomas

#### Overview of the analysis

**Fig. 5.**
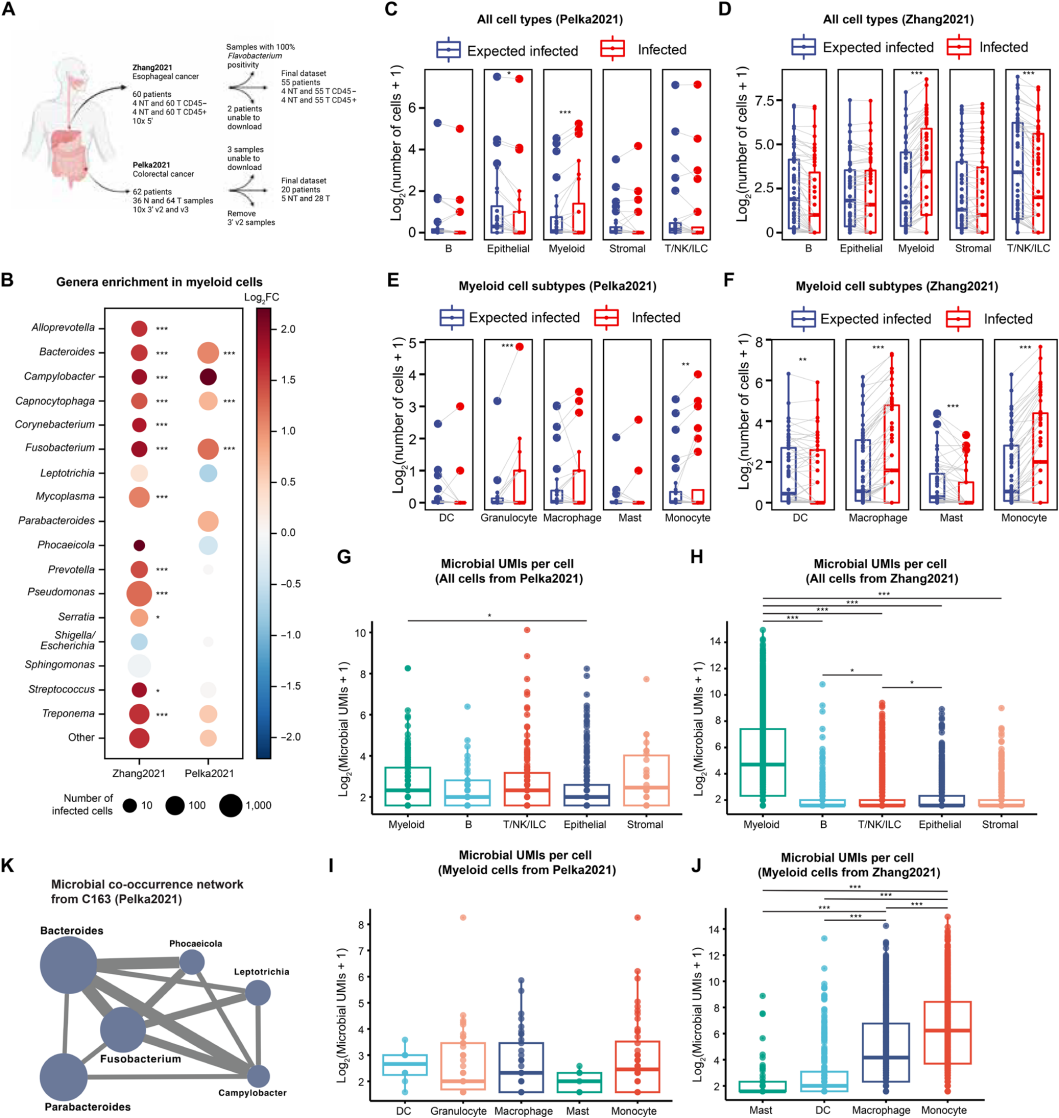
The cell type–specific bacterial landscape of CRC and ESCA patient tumors. (**A**) Overview of the samples analyzed in Pelka2021 and Zhang2021 scRNA-seq datasets. (**B**) The number of cells infected (≥2 UMIs) by each microbial genera where node size is relative to the number of infected cells (node sizes are normalized by the number of cells in each dataset), and node color indicates the log_2_FC in myeloid cells (>0 indicates enrichment in myeloid cells). We imposed a threshold of ≥10 infected cells to focus on the subset of genera seen in the highest number of cells. Complete unfiltered results are found in table S2. (**C** and **D**) The number of infected cells compared to expectation per sample for the top-level cell types from Pelka2021 (C) and Zhang2021 (D). (**E** and **F**) The number of infected cells compared to expectation per sample for the myeloid cell subtypes from Pelka2021 (E) and Zhang2021 (F). (**G** and **H**) The number of total microbial UMIs identified per infected cell by main cell type from Pelka2021 (G) and Zhang2021 (H). (**I** and **J**) The number of total microbial UMIs per cell by myeloid cell subtypes from Pelka2021 (I) and Zhang2021 (J). (**K**) The intracellular microbe co-occurrence network from colorectal tumor C163. Node size is relative to the number of cells infected by each microbe (Bacteroides is the largest node with *n* = 160 infected cells). Edges appear between microbes that co-occur in the same cell more than expected (FDR < 0.05), and edge width is proportional to the statistical significance of the co-occurrence. *P < 0.05; **P < 0.01; ***P < 0.001. For statistics used to determine DEGs and GSEA, see the Host cell transcriptomic analysis and Gene set enrichment analysis and visualization subsections of Materials and Methods.

Having demonstrated that our pipeline correctly and specifically identified reads from *Fusobacterium* in a controlled experiment, we next sought to examine the intracellular tumor microbiome of patient tumors in publicly available datasets. We decided to focus on colorectal and esophageal cancers because there are available 10x 3’ v3 and 5’ scRNA-seq datasets with large numbers of patients; *Fusobacterium* has been previously reported to be intracellular when present in tumors, which is not necessarily the cause for other bacteria reported in tumor samples and is associated with worse prognosis in both cancer types. Furthermore, the tumor microbiome of these cancer types has not been previously explored by scRNA-seq (the INVADE-seq paper included microbiome analysis of colorectal carcinoma using spatial transcriptomics and proteomics but not scRNA-seq) (*9*, *10*, *29*, *33*–*35*). The colorectal carcinoma cohort (a subset of the above Pelka2021 dataset) includes 30 tumor samples and 4 adjacent nontumor tissue samples from 20 tumors sequenced using 10x 3’ v3, after filtering out 10x 3’ v2 samples to their separate clustering, low number of microbial UMIs, and high number of *Mycoplasma* UMIs, which is a known wet and dry laboratory contaminant (Fig. 5A and fig. S6) (*36*, *37*). The esophageal carcinoma cohort, Zhang2021, includes 110 tumor samples (divided into CD45-positive and -negative cells) and 8 adjacent nontumor tissue samples from 55 esophageal carcinomas sequenced using 10x 5′ (Fig. 5A).

Next, we analyzed the tumor samples and observed a consistent range of the percentage of infected cells across patients in Pelka2021 (mean, 0.7% and range, 0 to 8.2% of the total number of cells) and Zhang2021 (mean, 4.25% and range, 0 to 21.7%), except for six tumor samples from three patients in Zhang2021 in which nearly 100% of cells were infected predominately by *Flavobacterium* (fig. S7), a previously reported cell culture contaminant (*38*). After excluding these six contaminated samples, we found a total of 569 infected cells (0.7% of the cells sequenced) from Pelka2021 and 6550 infected cells (3.4% of the cells sequenced) from Zhang2021 (see fig. S8 for UMAP of cell types and microbial UMIs). Fewer cells from the adjacent nontumor tissue samples were infected compared to the matched tumor tissue samples in both datasets (0.1 and 1.3% of cells from the paired nontumor in Pelka2021 and Zhang2021, respectively). Comparison of paired nontumor and tumor tissue after filtering for 10x 3’ v3 samples in Pelka2021 and *Flavobacterium*-low samples in Zhang2021 revealed no change in taxa diversity, microbial load, or number of infected cells (fig. S9). Overall, we found 91 genera that infect at least one cell in either dataset (table S2). Setting a threshold of ≥10 infected cells, we identified 17 common genera, including 10 genera found in both datasets, 6 genera specific to Zhang2021, and 1 genus specific to Pelka2021 (Fig. 5B).

#### Microbial UMIs are enriched in myeloid cells compared to other cell types in the TME

Given our previous in vitro finding that the proportion of bacterial read–positive cells can differ significantly between cell lines (Fig. 2D), we next asked whether specific cell types in the TME are also disproportionately infected. We found that myeloid cells were the only cell type with significantly more infected cells than expected in both Pelka2021 (*P* = 2 × 10^−10^; log_2_FC = 0.9) and Zhang2021 (*P* < 1 × 10^−300^; log_2_FC = 1.1); other cell types may have absolute or relative differences, but they were not statistically significant (Fig. 5, C and D, and Materials and Methods). Within the myeloid cell compartment, monocytes were the only myeloid cell subtype with bacteria engulfed significantly more than expected in both Pelka2021 (log_2_FC = 0.88; *P* = 0.006) and Zhang2021 (log_2_FC = 1.65; *P* = 5 × 10^−247^) (Fig. 5, E and F). This enrichment in myeloid cells was not due to a small number of genera, as 15 of 17 of the commonly found genera and all other genera combined are found in myeloid cells more than expected in at least one dataset (Fig. 5B and table S2). This enrichment in myeloid cells is robust for all UMI cutoffs analyzed (one to five microbial UMIs) in both Pelka2021 and Zhang2021 (table S2).

Next, we compared the number of microbial UMIs per infected cell across cell types and found that myeloid cells with bacteria engulfed contained significantly more microbial UMIs compared to infected nonmyeloid cells in Zhang2021 (*P* < 1 × 10^−300^) but not Pelka2021 (Fig. 5, G and H). Within the myeloid cell compartment, monocyte cells with bacteria engulfed contained significantly more microbial UMIs than the other myeloid cell subtypes in Zhang2021 but not in Pelka2021 (Fig. 5, I and J). These results showcase the importance of myeloid-derived cells, particularly monocyte cells, as the predominant source of bacterial RNA in the intracellular tumor microbiome.

#### Bacterial genera exhibit specific co-occurrence relationships in the same cells

We observed that many more cells were infected by multiple bacterial genera than expected by chance alone (64 versus 18 in Pelka2021; 205 versus 108 in Zhang2021). This was true for both myeloid cells (10 versus 3.5 in Pelka2021; 134 versus 91 in Zhang2021) and nonmyeloid cells (54 versus 15 in Pelka; 64 versus 34 in Zhang2021) in both datasets. To evaluate the possibility that these coinfected cells are the same cell-type doublets, we compared the number of (human gene) UMIs between coinfected and single infected cells. We observed significantly more UMIs in coinfected cells compared to infected cells in Zhang2021 (Wilcoxon rank sum test *P* = 0.006) albeit with a moderate increase (9058 versus 7851 median UMIs) and did not observe any statistically significant difference in Pelka2021 (Wilcoxon rank sum test *P* = 0.72). We conclude that there may be some doublets in the Zhang2021 dataset that the original authors failed to detect, but those doublets are not a serious problem in the Pelka2021 dataset. As specific bacterial genera including *Fusobacterium*, *Leptotrichia*, and *Campylobacter* have been described as co-enriched in colorectal tumors (*39*, *40*), we examined whether co-infection relationships between specific bacterial genera could be detected within single cells of individual tumors (Materials and Methods). In total, we found 12 significant coinfection relationships in Pelka2021 and 9 significant coinfection relationships in Zhang2021 (FDR-corrected *P* < 0.05; table S3, which contains separate sheets for the two datasets). Occasionally, we observed infection of the same cell by three or more bacterial genera (*n* = 19 and *n* = 15 in Pelka2021 and Zhang2021). One extreme example of this phenomenon is colorectal tumor C163 in which we found 14 cells coinfected by three or more genera, including a single tumor cell positive for six genera, including *Fusobacterium* (150 UMIs), *Campylobacter* (96 UMIs), and *Bacteroides* (42 UMIs) (Fig. 5K). Our results suggest that bacterial genera may preferentially coinfect the same single cells in the TME, which agrees with a previous report that primary *F. nucleatum* infection greatly increased secondary invasion rates of other species in vitro (*41*). The enrichment of coinfected cells is robust to the UMI threshold (one to five microbial UMIs) in both Pelka2021 and Zhang2021 (table S3).

#### Intracellular bacteria induce the up-regulation of antigen presentation genes in infected host tumor cells but their down-regulation in infected myeloid cells

Next, we sought to identify any host transcriptomic changes associated with the presence (or engulfment) of intracellular bacteria in host cells. We first examined principal components (PCs) between uninfected and infected cells to determine whether any global transcriptomic changes were associated with infection (Materials and Methods). We observed that most of the five PCs were significantly different between infected and uninfected cells for most cell types in both Pelka2021 and Zhang2021 (table S4). This effect was most prominent in epithelial and myeloid cells, where at least four of five PCs were significantly different in both datasets. We then performed differential expression analysis between infected and bacterial-negative cells for each cell type and each dataset separately. We observed significant overlap between the differentially expressed genes (DEGs) identified using UMI ≥ 2 and those identified using the thresholds ≥1, 3, 4, and 5 UMIs demonstrating the robustness of the DEGs to the microbial UMI threshold (table S4). Intriguingly, we found that myeloid cells and tumor cells had many DEGs between infected and bacterial-negative cells (means, 396 and 390 DEGs for myeloid and tumor cells, respectively) compared to few DEGs in T/natural killer (NK)/innate lymphoid (ILC) cells (mean, 49 DEGs) (table S4). We found 286 DEGs in stromal cells compared to only 19 DEGs in B cells in Zhang2021 but only two and four DEGs in stromal and B cells in Pelka2021. Subsampling analysis to control for cell number identified very few DEGs in any cell type in Pelka2021 using the number of infected and uninfected stromal cells, suggesting that the low number of DEGs is due to cell number. In contrast, the number of DEGs for all cell types in Zhang2021 and myeloid, epithelial, and T/NK/ILC cells in Pelka2021 was consistent across subsampling. We also observed substantially more DEGs for live-*Fn*–exposed HCT116 epithelial cells (571 and 156 DEGs compared to unexposed and HK-*Fn*–exposed cells, respectively) and THP1 monocytic cells (1985 and 249 DEGs) compared to Jurkat T cells (143 and 61 DEGs) from Robinson2023-10x (table S4). Given these results, we focused our analysis on myeloid and tumor cells and performed gene set enrichment analysis (GSEA) (*42*) on the DEGs identified between infected and bacterial-negative cells. We further clustered gene sets with overlapping genes using EnrichmentMap (*43*, *44*).

**Fig. 6.**
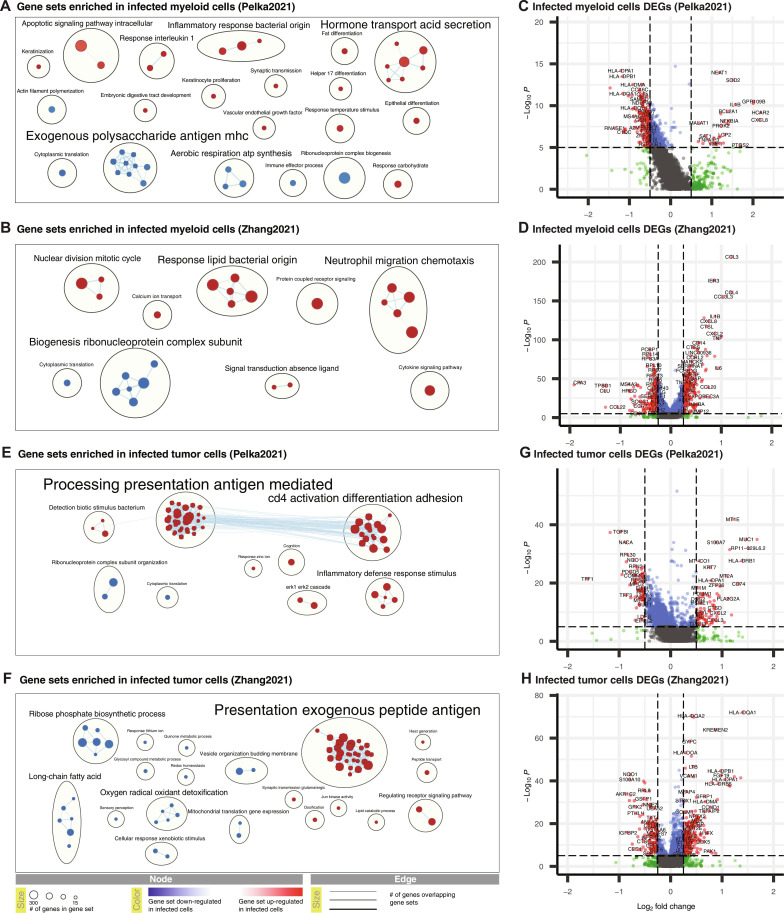
Host cell transcriptomic changes associated with bacterial infection. (**A**) EnrichmentMap of clustered gene ontology (GO) biological processes (BP) gene sets up and down-regulated in infected myeloid cells from Pelka2021. (**B**) EnrichmentMap of clustered GO BP gene sets up- and down-regulated in infected myeloid cells from Zhang2021. (**C**) Volcano plot showing differentially expressed genes (DEGs) between infected and bacterial-negative myeloid cells from Pelka2021. (**D**) Volcano plot showing DEGs between infected and bacterial-negative myeloid cells from Zhang2021. (**E**) EnrichmentMap of clustered GO BP gene sets up- and down-regulated in infected tumor cells from Pelka2021. (**F**) EnrichmentMap of clustered GO BP gene sets up- and down-regulated in infected tumor cells from Zhang2021. (**G**) Volcano plot showing DEGs between infected and bacterial-negative tumor cells from Pelka2021. (**H**) Volcano plot showing DEGs between infected and bacterial-negative tumor cells from Zhang2021. For statistics used to determine *P* values, see Calculation of number of expected infected cells per cell type (10x Genomics protocols) and Calculation of co-infection relationships in colorectal cancer and esophageal cancer datasets subsections of Materials and Methods.

Myeloid cells with bacteria engulfed in both datasets shared a cluster of up-regulated gene sets associated with response to molecules of bacterial origin, which points to the potential and consistent functional impact of these bacterial UMIs (Fig. 6, A and B, and table S5). We previously observed that monocytes disproportionately engulf bacteria relative to other myeloid cells in both datasets (Fig. 5, E and F), so we repeated our analysis comparing monocytes with bacteria engulfed to bacteria-negative monocytes and found similar enriched pathways (fig. S10, A and B), which suggests that the identified enriched pathways are not due to different myeloid subtype specificities.

At the individual gene level, 15 genes were up-regulated in infected myeloid cells in both datasets (>50-fold over-enrichment; *P* < 1 × 10^−25^), and 33 were down-regulated in both datasets (>8-fold over-enrichment; *P* < 1 × 10^−20^) (Fig. 6, C and D; fig. S10, C and D; and table S5). Repeating this analysis, shuffling the infection labels (50 times using a specified seed), identified zero DEGs in 96 and 100% of the iterations in the Pelka2021 and Zhang2021 datasets, respectively, and consequently zero overlapping DEGs, testifying to the statistical significance of the identified overlap. These 15 up-regulated genes shared by Pelka2021 and Zhang2021 strongly overlapped with both the set of up-regulated genes in THP1 cells exposed to live *F. nucleatum* in Robinson2023-10x (12-fold over-enrichment; *P* < 4 × 10^−13^) and the set of genes with nuclear factor kappa B (NF-κB) promoter sites (NF-κB target genes) (23-fold enrichment; *P* = 1 × 10^−12^) (https://bu.edu/nf-kb/gene-resources/target-genes/) (fig. S10C and table S5). NF-κB can be activated by several up-stream mechanisms including Toll-like receptors, which recognize conserved microbial molecules (pathogen-associated molecular patterns). Of the 10 NF-κB target genes up-regulated in all datasets, *CXCL8* is potentially the most relevant clinically because high levels of interleukin-8 (IL-8) (the protein encoded by *CXCL8*) in the serum and the TME have been associated with a reduced clinical benefit from immune-checkpoint inhibitors across multiple cancer types (*45*, *46*). One of these previous reports found that intratumoral IL-8 production was driven primarily by *CXCL8-*high myeloid cells that up-regulated IL-1 response genes (observed in both datasets) (Fig. 6, C and D, and fig. S11) (*46*). Combining these findings suggests that myeloid cells with bacteria engulfed in the TME causes the production of IL-8, which decreases response to immune checkpoint blockade, a hypothesis for future experimental investigation.

Beyond the binary differential expression analysis, we further hypothesized that the actual number of microbial UMIs per cell would be a direct function of the number of infecting microbes, and these numbers may be associated with host cell gene expression changes. By analyzing gene expression at the single-cell level and averaging over cells, we identified genes whose expression significantly correlated with the number of microbial UMIs per cell in myeloid cells (excluding bacteria-negative myeloid cells) in both datasets (table S6). Reassuringly, we found the rho values for dysregulated genes to be significantly correlated between Pelka2021 and Zhang2021 (Spearman rank correlation rho = 0.14, *P* < 2 × 10^−58^) and observed that previously discussed genes were positively correlated (*CXCL8* and *IL1B*) with microbial load in both datasets. We then repeated this analysis using only one genus (*Pseudomonas*, which is the most abundant genus in Zhang2021) and identified similar results (table S6). In addition, we provide a table of all significant associations between microbial load and gene expression for all 17 common genera and all cell-types in both datasets (table S6). These results emphasize “microbial load” as an important factor in modulating host cell gene expression, something to be considered in future scRNA-seq–based explorations of the tumor microbiome.

Next, we analyzed tumor cells and observed the largest cluster of up-regulated gene sets in infected tumor cells to be associated with antigen processing and presentation and cell killing by leukocytes in both Pelka2021 and Zhang2021 (Fig. 6, E to H). At the individual gene level, we found 23 genes up-regulated in infected tumor cells in both cancer types (13-fold over-enrichment; *P* < 1 × 10^−19^), including the cytoskeletal gene *KRT7*, which is up-regulated by *F. nucleatum* infection and promotes metastasis of colorectal carcinoma (*47*), and 12 genes involved in antigen processing and presentation of peptides on human leukocyte antigen (HLA) class I and class II (see fig. S10, E and F for the complete list). The up-regulation of HLA genes by infected tumor cells is unexpected on two levels. First, intracellular bacteria have been reported to decrease HLA expression levels in host cells to evade the immune system (*27*). Second, tumor cells also have frequent HLA alterations including HLA loss of heterozygosity (*48*, *49*) and HLA down-regulation (*50*) to evade the immune system. While the idea that tumor cells and intracellular bacteria may cooperate to evade the immune system together is intuitive, our transcriptomic data suggest that tumor cells respond to intracellular bacteria by up-regulating antigen processing and presentation, which may increase the presentation of neo-antigens and lead to tumor killing by the immune system.

Collectively, our analyses of the proportion of infected cells, the number of microbial UMIs per infected cell, and host transcriptomic changes associated with infection from patient tumor scRNA-seq datasets suggest a model of the tumor microbiome. In this model, bacteria predominately reside within or are engulfed by myeloid cells that trigger inflammatory pathways like tumor necrosis factor and IL-1 via NF-κB pathway activation. In contrast, while bacterial UMIs are strongly enriched in tumor samples (compared to nontumor adjacent tissue), they are not enriched in tumor cells by either the proportion of infected cells or the number of microbial UMIs per infected cell. One potential explanation for this is the up-regulation of HLA genes in infected tumor cells, which suggests that tumor cells respond to bacterial invasion by “raising the alarm” even at risk of being detected and killed by the immune system.

## DISCUSSION

In this study, we sought to analyze intracellular microbes in cancer via the analysis of microbial reads from scRNA-seq data. Our first main goal was a methodological one—to systematically study the detection capabilities of different sequencing platforms compared to orthogonal approaches for studying intracellular bacteria. Because of the lack of suitable published positive control datasets, we performed in vitro *F. nucleatum* infection of cell lines of distinct lineages, which we validated using RNAscope and CFU counting and sequenced using both plate- and droplet-based scRNA-seq approaches. By analyzing this dataset (Robinson2023) along with other related publicly available datasets, we systematically identified important differences in the number of bacterial reads sequenced from known intracellular microbes and contaminants, both between plate and droplet-based approaches and, further, within droplet-based approaches (10x 3’ v2 vs. 3’ v3 and 5’).

The difference in microbial reads across 10x chemistries sheds further light on the distinct approaches to contamination taken by alternative existing scRNA-seq microbiome pipelines like SAHMI and INVADE-seq. SAHMI analysis of 10x 3’ v2 pancreatic datasets would have necessitated multiple decontamination protocols (*19*), given the relatively poor microbial read recovery of this assay. While the INVADE-seq study does not discuss contamination, it sets a comparable threshold (≥ 3 UMIs versus ≥ 2 UMIs, respectively) for host cell differential expression analysis for their 10x 5’ assay custom modified to detect bacterial 16S rRNA transcripts (*21*). Our analysis suggests an intermediate stringency with which unmodified 10x 3’ v3 and 5’ datasets (many of which have already been generated) can be analyzed for cell host–associated microbes using a simple threshold for removing contaminants and bystander cells.

On the basis of the findings related to our first, methodological analysis, we turned to our second, thematic goal: analyzing the intracellular microbiome of esophageal and colorectal carcinoma, to answer two basic questions: (a) What is the cell type–specific tumor microbiome landscape in these tumors? (b) What are the cell type–specific host transcriptomic changes that are associated with these infections? In response to the first question, we find that microbial UMIs can be found predominately (but not exclusively) within myeloid cells in the TME and that some intracellular bacteria co-occur within the same cells in the TME. Answering the second question, we find that myeloid cells with bacteria engulfed and infected tumor cells have cell type–specific transcriptomic alterations with potential functional impact.

Our analysis of the host transcriptomic changes in infected cells suggests that bacteria may play an important role in modulating immune processes, which suggests that intratumoral microbial load may affect the efficacy of cancer immunotherapy. Myeloid cells with bacteria engulfed appear to up-regulate expression of *CXCL8* (and likely production of IL-8), and high levels of IL-8 have been reported to be associated with decreased response to immune checkpoint blockade (*46*). In contrast to myeloid cells with bacteria engulfed, infected tumor cells transcriptionally up-regulate antigen processing and presentation and other pathways that may make them more susceptible to killing by T cells. These results suggest that not only microbial load, but also type of infected cell, are important considering factors in designing immunotherapy regimens.

There are several important limitations of our study, most notably our hypothesis that the microbial UMIs we identified across scRNA-seq datasets are indicative of viable intracellular bacteria in nonmyeloid cells. First, although we show multiple lines of evidence that 10x 3’ v3 appears to better capture bacterial RNA compared to 10x 3’ v2, we lack the gold standard comparison of the same sample exposed to an intracellular bacterium and sequenced using both technologies because 10x had already discontinued the 10x 3’ v2 kit when we performed our in vitro infection experiments. Second, our arbitrary cutoff of ≥2 UMIs may not be sufficient to distinguish infected from bystander cells. Third, one cannot rule out that at least some of the polymicrobial communities we describe as intracellular are actually extracellular but adhere to the cell surface via biofilm formation. Polymicrobial biofilms featuring *Fusobacterium* and *Bacteroides* have been described in colorectal cancer (*51*). Fourth, it is also possible that some of the RNA reads may emanate from vesicles, such as outer membrane vesicles, either freely circulating or merged with eukaryotic cells, and are misattributed to being intracellular bacteria in our analyses. Fifth, microbial contaminants may be introduced during laboratory manipulation of tissue samples, which we find evidence of in the esophageal dataset. Nearly 100% of cells showed infection in three patients, and the identified genus was common between all three samples. Although this is, to the best of our knowledge, the first report of contamination within scRNA-seq data, we note that laboratory contamination has been recognized as an important issue in cell cultures with implications for cell phenotype and biology (*11*).

In summary, we have introduced a computational pipeline and scRNA-seq dataset, which together enabled a systematic evaluation of multiple scRNA-seq technologies for their efficacy in recovering microbial UMIs from intracellular bacteria and contaminants. We found that 10x 3’ v3 and 5’ chemistries are superior to both earlier chemistries and plate-based approaches. To demonstrate the utility of our pipeline and this finding, we analyzed two scRNA-seq cancer datasets, which identified the enrichment of bacterial UMIs in myeloid cells and the association of bacterial UMIs with cell type–specific gene expression signatures.

## MATERIALS AND METHODS

### Experimental design

This study aimed to investigate whether reads from intracellular bacteria could be detected (and distinguished from contaminants) using scRNA-seq technologies (and which technology would perform best) to explore the intracellular tumor microbiome. To answer our first question, we built a computational pipeline to analyze microbial reads from scRNA-seq technologies. Next, we first set up an in vitro infection model system using the intracellular bacterium *F. nucleatum* and three cell lines (THP1, Jurkat T, and HCT116), which we validated using CFU counting and RNAscope. We included multiple cell lines of different cell type origin to investigate whether bacterial invasion rates differed by cell type. Next, we performed scRNA-seq using three technologies (Seq-Well plexWell, 10x 3’ v3, and 10x 5’ v2) on cells exposed in vitro to *F. nucleatum* and appropriate controls. We analyzed the results from these datasets in conjunction with publicly available scRNA-seq datasets and decided that 10x 3’ v3 and 10x 5’ were the best technologies for analyzing the intracellular tumor microbiome. We identified and analyzed two large published scRNA-seq datasets of colorectal and esophageal carcinoma sequenced using 10x 3’ v3 and 5’. We investigated and found enrichment of bacterial UMIs in specific cell types (myeloid-derived cells) and found host cell DEGs associated with bacterial UMIs. Unexpectedly, we also noticed that different bacterial genera were found in the same host cells more than expected by chance alone.

### Cell and bacterial culture

HCT116 (colorectal carcinoma) cells were grown in McCoy’s 5A media containing 10% fetal bovine serum (FBS, Corning, #10-050-CV). Jurkat T (T cell acute lymphoblastic leukemia) and THP-1 (acute monocytic leukemia) cells were each grown in RPMI 1640 containing 10% FBS (Corning, #10-040-CV). All cell lines were maintained in a 37°C incubator with 5% CO_2_. *F. nucleatum* [American Type Culture Collection (ATCC) 25586] was cultured anaerobically in Columbia broth (BD Difco, #294420) at 37°C with 200-rpm shaking. All cell lines and bacterial strains were obtained from the ATCC (Manassas, VA). Cell line identities were authenticated by the ATCC.

To reduce the possibility of cell line contamination, we performed the following QC steps: (i) All cell work was handled in a biological safety cabinet under biosafety level 2 conditions; (ii) cells were routinely and periodically tested for *Mycoplasma* spp. contamination; (iii) we detected only one *Mycoplasma* spp. UMI by CSI-Microbes; and (iv) bacterial invasion was verified by colony counting on blood agar, which returned only one colony morphology (consistent with *F. nucleatum*) after incubation with *F. nucleatum* and no colony growth from unexposed cells or cells exposed to heat-killed *F. nucleatum*.

### Invasion assay

Cell lines were seeded at a density of 10^6^ before infection. Bacteria were grown to late log phase, and then 5 μM CellTrace Violet (Thermo Fisher Scientific, #C34571) was added to the broth culture for 20 min with shaking. Bacteria were pelleted and washed in sterile 1× phosphate-buffered saline (PBS, Corning, #21-031-CV) and then added to cell culture media at a multiplicity of infection of 100:1. Cells were infected for 10 hours, and then the medium was removed and cells were washed twice with 1× PBS. Fresh media containing gentamicin (200 μg/ml; Thermo Fisher Scientific, #15750060) and metronidazole (200 μg/ml; Thermo Fisher Scientific, #AC210340050) were added to the cells for 1 to 2 hours to kill extracellular bacteria. After antibody incubation, cells were washed twice with sterile 1× PBS and then collected for downstream analysis by trypsinization (HCT116 only). Bacterial invasion efficiency was determined by serial dilution and plating on trichostatin A + 5% sheep’s blood (Remel, #R01200) following a 10-min incubation of cells in 1% Triton X-100 (Sigma-Aldrich, #T8787). Agar plates were incubated anaerobically for 72 hours before colony enumeration.

### RNAscope analysis

Following bacterial infection and antibiotics treatment as described above, cells were washed twice with sterile 1× PBS and then resuspended in 25 μl of human plasma. Next, 25 μl of thrombin was added and mixed by pipetting and then allowed to clot for 1 to 2 min. Clots were fixed overnight in 10% formalin (Thermo Fisher Scientific, #032-059) and then sectioned at 5-μm thickness. RNAscope 2.5 LSx Reagent Kit-RED (ACD, #322750) was used according to the manufacturer’s protocol using the Leica BOND RX instrument with probe against *F. nucleatum* 23*S* rRNA (ACD, #485418). The number of positive cells was evaluated by a pathologist on a brightfield microscope.

### Droplet-based single-cell partitioning, library preparation, and sequencing (10x Genomics)

Single-cell suspensions were prepared and concentration and viability measured on an automated dual-fluorescence cell counter with acridine orange and propidium iodide stain (Luna Fx7, Logos Biosystems). Single-cell partitioning and RNA-seq library preparation was performed using 10X Genomics Chromium NextGEM 5’ v2 chemistry (user guide CG000331) or 10X Genomics 3’ v3.1 chemistry (user guide CG000204) according to vendor recommendations. Sample viability was above 80% for all conditions. Six to eight thousand cells were targeted to be captured for each sample. Libraries were sequenced on the NovaSeq 6000 with a target depth of 50,000 reads per cell using read parameters recommended by 10x Genomics user guides. Reads from multiplexed sequencing runs were demultiplexed using cellranger mkfastq v7.0.0 (10x Genomics).

### Plate-based single-cell partitioning, library preparation, and sequencing (SeqWell PlexWell)

Single-cell suspensions were prepared and sorted on a BD FACS Aria IIU into 96-well polymerase chain reaction plates that were prepared with lysis buffer according to seqWell plexWell Rapid Single-Cell method (user guide v20210402) and containing ERCC spike-in mix (Invitrogen # 4456740) at a dilution of 1:1E7. Following deposition of single cells into prepared plates, they were snap-frozen and stored at −80C until further processing. Single-cell cDNA and libraries were generated for each sorted sample according to seqWell plexWell Rapid Single-Cell user guide. Multiplexed libraries containing indexed scRNA-seq libraries were sequenced on the either the NextSeq 2000 or NovaSeq 6000 with a target read depth of 2 million reads per cell using the following read parameters: read 1, 61 base pairs (bp); index 1, 8 bp; index 2, 8 bp; and read 2, 61 bp. Raw sequencing data were demultiplexed into individual sample fastq sets using bcl2fastq v2.20.0.

### Plate-based single-cell dataset alignment to the human genome and downstream analysis

Raw FASTQ files were trimmed using fastp (*52*) v0.20.1 with the arguments “*--unqualified_percent_limit 40 --cut_tail --low_complexity_filter --trim_poly_x*”. The trimmed FASTQ files were aligned to the reference human genome (GRCh38 gencode release 34) and any applicable spike-in sequences using STAR (*53*) 2.7.6a_patch_2020-11-16 with the arguments “--soloType SmartSeq --soloUMIdedup Exact --soloStrand Unstranded --outSAMunmapped Within.” For analysis, we used the previously described workflow “Lun 416B cell line (Smart-seq2)” from “Orchestrating Single-Cell Analysis with Bioconductor” (http://bioconductor.org/books/3.16/OSCA.workflows/lun-416b-cell-line-smart-seq2.html#lun-416b-cell-line-smart-seq2) to specifically handle ERCC spike-in sequences. Quality filtering was performed using the quickPerCellQC function from scuttle (*54*) v1.8.0.

### 10x single-cell dataset alignment to the human genome and downstream analysis

Raw FASTQ files were aligned to the reference human genome (GRCh38 Gencode release 34) using CellRanger (*55*) v5.0.1 (https://support.10xgenomics.com/single-cell-gene-expression/software/pipelines/latest/what-is-cell-ranger). The annotated polyA and template sequence oligonucleotide sequences were trimmed, the unmapped reads were converted to the FASTQ file format trimmed and filtered using FASTP as described above before being converted to BAM files. For quality filtering, we removed genes found in fewer than three cells and removed cells with fewer than 3000 gene features or greater than 15% of reads mapping to mitochondrial genes.

### Annotation of cell types

For the Plex-Well dataset generated by this study, we sorted cells with known identity (HCT116 or Jurkat T) into wells or intentionally left wells empty. For the 10x datasets, we performed low-resolution clustering using Seurat (resolution = 0.1) and annotated clusters using marker genes for HCT116 (*EPCAM*), Jurkat T (*CD3E*), and THP1 (*CD64*/*FCGR1A*).

For previously generated datasets, we used the cell type annotations provided by the original authors. We syntactically harmonized annotations between the esophageal (Zhang2021) and colorectal (Pelka2021) datasets as follows. From the esophageal dataset, we grouped endothelial, FRC, fibroblast, and pericytes as stromal cell (the top-level annotation for these cell types used in the colorectal carcinoma dataset). From the colorectal dataset, we grouped plasma cells as B cells and mast cells as myeloid cells using the author-supplied top-level annotations. The cell-type annotation harmonization resulted in five major cell-type groupings: epithelial (tumor), stromal, B, T/NK/ILC, and myeloid.

### Alignment of unmapped reads to microbial genomes

The unaligned reads were assigned to microbial taxa using PathSeq (*23*) v4.1.8.1 (http://software.broadinstitute.org/pathseq/) with the arguments “*--filter-duplicates false --min-score-identity .7.*” We constructed the reference microbial genome database by downloading the set of complete viral, bacterial, and fungal genomes from RefSeq release 201 (*56*). We subsampled at least one genome from each species including any genomes annotated as either “reference genome” or “representative genome” as well as the genomes of the three *Salmonella* strains used in the analyzed datasets. To mitigate vector contamination, we identified regions of suspected vector contamination (including “weak” matches) in the genomes using Vecscreen_plus_taxonomy (https://github.com/aaschaffer/vecscreen_plus_taxonomy) with the UniVec database (ftp://ftp.ncbi.nlm.nih.gov/pub/UniVec/) and filtered any reads that aligned to these regions (*57*).

PathSeq uses alignment to a finite database of reference genomes. As explained in the Results section entitled “CSI-Microbes and INVADE-seq provide improved sensitivity in microbial read identification compared to SAHMI,” the PathSeq alignment parameters can be stringent and INVADE-seq sets one key parameter even more stringently than CSI-Microbes. The stringent parameter setting is sensible in a setting where one does not have prior knowledge about which microbial taxa are expected. The experimental setup in our analysis of Robinson2023-plexWell, for example (table S1A), is different in that we expect to find reads from the genus *Fusobacterium*. Therefore, we did a targeted assessment of which reads counted in table S1A as mapped to non-*Fusobacterium* could plausibly originate from *Fusobacterium* and could be detected as such if one used a more permissive alignment method. Specifically, we used blastn (*58*). The blastn alignments sought could have been missed by PathSeq either because the PathSeq internal settings were too stringent or because the coverage of *Fusobacterium* diversity was insufficient in PathSeq’s database of reference genomes. We did the main search with the non-defautlt blastn command:

blastn -query <input sequence file>.fa -db Fuso -outfmt 6 -out <output sequence file>.out -evalue 1e-15 -perc_identity 95 -word_size 11.

Eleven is the smallest permitted value of word_size that defines the minimum length of the initial seed alignment, which is later extended and hence is the most permissive word size. Here, Fuso is a database of 16 *Fusobacterium* genomes (table S1D), which contains genomes in PathSeq database and other genomes. To find useful *Fusobacterium* genomes to add to the blastn database Fuso, we i) sampled sequences that had much less significant matches to the PathSeq *Fusobacterium* genomes and then ii) queried those sequences against National Center for Biotechnology Information (NCBI’s) databases of genomes with default web-blastn parameters to find better alignments to other *Fusobacterium* genomes. Not all the 16 genomes used are from the species *F. nucleatum*, which is the infecting microbe used in our wet laboratory experiment. Thus, the sequences found by blastn may not be real matches. They represent a cautious upper bound on how many sequences in table S1A classified as non-*Fusobacterium* may be false positives.

### Comparison of CSI-Microbes with INVADE-seq and with SAHMI

We ran two alternative pipelines (INVADE-seq and SAHMI) for the detection of microbial reads from scRNA-seq datasets on Robinson2023-10x (*19*, *21*). We used the same microbial database files as described above and used for CSI-Microbes. The exact code used to run these pipelines is available on the CSI-Microbes-identification GitHub and explained in the README.md. Specifically, the code used to run SAHMI is in the file run-SAHMI.smk in the Robinson2023-10x directory (https://github.com/ruppinlab/CSI-Microbes-identification), and the code to run INVADE-seq is in the file run-SAHMI.smk in the Robinson2023-10x directory (https://github.com/ruppinlab/CSI-Microbes-identification).

At a high level, we used the shell commands in https://github.com/FredHutch/Galeano-Nino-Bullman-Intratumoral-Microbiota_2022/blob/37049d3d8e7f78f09a1f5dc505e5544b9b503434/patient_samples/patient_samples_GEX_pipeline.sh for INVADE-seq and converted them to a file of steps for snakemake. SAHMI provided a README (but not a shell script) that specified the functions to run, which we used to generate the set of commands for snakemake. For the outcomes reported above in Results, we included all of the steps except for the sample k-mer correlation test function due to the small number of samples (*n* = 4 in Robinson2023-10x) and because each sample constituted a separate experimental condition. To check whether including the sample k-mer correlation test would substantially change the results, we ran it on all four samples together and found only two (spurious) species (*Acinetobacter cumulans* and *Georgenia faecalis*) and zero genera were significantly (*P* < 0.05) and positively (*r* > 0) correlated across samples.

### Statistical analysis

#### Cell type enrichment (plate-based protocols)

We define the abundance of a particular microbe in each cell to be the number of reads assigned unambiguously to the relevant genome(s) by PathSeq. The abundances are normalized using the computeSpikeFactors function from scran (*59*) v1.16.0 (https://github.com/MarioniLab/scran), which computes the library size factors using the sum of the spike-in sequences. To limit the number of hypotheses, we only test microbial taxa with counts per million microbial reads >10 in at least 50% of the cells from a cell type. The logged normalized read counts are compared across cell types using the findMarkers function from scran v1.16.0 with arguments “*test*= ‘*wilcox*,’ *lfc*=*0.5*, *block*= ‘*plate*’”. The findMarkers function from scran v1.16.0 makes inconsistent assumptions about how to distribute the values of the null distribution depending on whether the user specifies “*direction* = ‘*up*’” or “*direction* = ‘*down*’” (a one-sided test) or the user specifies “*direction* = ‘*any*’” when the parameter *lfc* is greater than zero (https://github.com/MarioniLab/scran/issues/86). The assumption for the one-sided test models our intent, so we ran the comparison twice, once using with “*direction* = ‘*up*’” and once with “*direction* = ‘*down*,’” selected the result with the smaller *P* value for each microbial taxa, and converted the one-sided *P* value to the two-sided *P* value by taking the minimum of 1 and 2*P value as described in a standard reference (*60*) (page 79).

#### Cell type enrichment (10x Genomics protocols)

The analysis of microbial reads in single-cell sequencing data is a relatively new area with few established statistical methods. For this reason, we chose to not make any assumptions about the underlying distribution of microbial reads in scRNA-seq. Following this decision, we chose to use nonparametric statistical tests. We observed from our in vitro infection experiments that most cells sequenced by the 10X platform contained zero bacterial UMIs. Therefore, we decided to categorize cells as either infected or uninfected using a threshold of ≥ X UMIs assigned unambiguously to the relevant genome(s) by PathSeq and to use a test of categorical data to calculate cell type enrichment. We chose Fisher’s exact test as our test of categorical data instead of Chi-squared approximation because “for the chi-square approximation to be valid, the expected frequency should be at least 5” (https://itl.nist.gov/div898/handbook/eda/section3/eda35f.htm), and this condition is not met for many sample, cell type, and genus combinations.

It is important to evaluate cell-type enrichment in individual samples instead of evaluating cell-type enrichment using all cells (and ignoring samples). We illustrate this using the following toy example with two samples (s1 and s2) and two cell types (ct1 and ct2)

s1: 100 cells; 80 cells are ct1, and 20 cells are ct2; 16 (20%) ct1 cells are infected, and 10 (50%) ct2 cells are infected.

s2: 100 cells; 20 cells are ct1, and 80 cells are ct2; 0 ct1 and 0 ct2 cells are infected.

Looking only at sample s1, ct2 is enriched in infected cells compared to ct1 (50 versus 20%). However, combining s1 and s2 would show that 16 (16%) of ct1 cells were infected compared to only 10 (10%) in ct2, leading to the erroneous conclusion that ct1 is enriched in infected cells.

We illustrate our approach by comparing the enrichment of infection by any genera in myeloid versus nonmyeloid cells in sample C169_T_1_1_0_c1_v3 from patient C169 from Pelka2021 using X = 2 as the threshold for infection. This sample had 2245 cells, of which 473 cells were myeloid and 1772 cells were nonmyeloid. We first compare the proportion of cells infected by a given microbial taxa between two cell types in a sample using the fisher_test function from SciPy.

For sample C169_T_1_1_0_c1_v3, 2.3% (51 of 2245) of cells are infected, including 5.5% (26 of 473) of myeloid cells and 1.4% (25 of 1772) of nonmyeloid cells. We calculate the *P* value using Fisher’s exact test (code and contingency table shown below), which is (rounded) equal to 1.68 × 10^−06^.

fisher_test([[26, 447], [25, 1747]], alternative = “greater”)

To calculate the effect size, we first calculate the number of expected infected cells per cell type under the null hypothesis using the formula

(number of cells from that cell type/number of cells) * number of infected cells

Using this formula, we calculate the number of expected infected myeloid cells to be 10.75 [473*(51/2245)] and the number of expected infected nonmyeloid cells to be 40.25 [1772*(51/2245)]. Comparing the number of infected myeloid cells to the number of expected infected myeloid cells yields 2.4-fold increase (26/10.75) or a 1.3 log_2_ fold increase [log_2_(26/10.75)] in the number of infected cells relative to expectation.

To calculate the log_2_ fold change for a given cell type across a cohort, we first sum the number of infected cells of that cell type across all samples. Next, we sum the number of expected infected cells of that cell type across all samples. Last, we calculate the log_2_ fold increase as shown above.

To calculate the significance of the infection enrichment for a particular cell type across a cohort, we first calculate the per sample *P* value for the given cell type using alternative = “greater” (we use alternative = “less” to calculate the significance of the infection depletion for a particular cell type). Next, we combine the per sample *P* values for a given cell type using Stouffer’s *Z*-score method (using the combine_pvalues function with argument `method = “stouffer”` from SciPy) weighting the *P* values using the number of expected infected cells (as samples with very few infected cells or very few cells of the cell type of interest will not be informative). We set the maximum per sample *P* value to be 0.9999 as combine_pvalues function with method = “stouffer” returns (-Inf, 1) when any *P* value = 1 (https://github.com/scipy/scipy/issues/8506).

### Calculation of coinfection relationships in colorectal cancer and esophageal cancer datasets

In each dataset (Pelka2021 and Zhang2021), we calculated coinfection relationships only for genera that infect ≥10 cells. We initially calculate coinfection relationships per sample. Given genera g1 and genera g2, we calculate the coinfection relationship using the hypergeometric enrichment to ask whether the number of cells infected by both g1 and g2 are more than expected knowing the total number of cells, the number of cells infected by g1, and the number of cells infected by g2 (using the hypergeom function from SciPy). Next, for a given coinfection relationship, we combine the per-sample *P* values using Stouffer’s *Z*-score method (using the combine_pvalues function with argument method = stouffer from SciPy) weighting the *P* values using the number of expected coinfected cells (as samples with very few infected cells of g1 or g2 will not be informative). We set the maximum per sample *P* value to be 0.9999 as combine_pvalues function with method = stouffer returns (-Inf, 1) when any *P* value = 1 (https://github.com/scipy/scipy/issues/8506).

### Host cell transcriptomic analysis

We performed differential gene expression analysis between infected (≥2 microbial UMIs) and bacterial-negative (0 microbial UMIs) human cells for myeloid and tumor cells. We used the function FindMarkers from Seurat (v4.3.0) using the default value for the host transcriptomic results.

### Principal components analysis

To identify PCs associated with infection status, we processed all cells from a particular cell type (i.e., myeloid) and dataset (i.e., Pelka2021) through Seurat (v4.3.0) up to and including the RunPCA function. Next, we extracted the values of the first five PCs for every cell using the function FetchData from Seurat (v4.3.0). Last, we compared the values for each PC between uninfected and infected (≥2 microbial UMIs) cells using the function ranksums from scipy.stats.

### GSEA and visualization

For GSEA, we performed differential gene expression analysis as described above using the additional argument of “logfc.threshold = -Inf, min.pct = -Inf, min.diff.pct = -Inf” to return a *P* value and avg_log_2_FC for every gene. We ranked genes using the formula: avg_log_2_FC*−log_10_(*P* value +1 × 10^−300^). We added 1 × 10^−300^ to avoid errors when the *P* value equaled to zero. We ran GSEAPreranked (*42*) (v4.3.2) using preranked genes (described above) and the Gene Ontology Biological Processes v.7.5.1 gene sets using the default parameters and seed = 0.

We visualized the enriched gene sets using Cytoscape (*61*) (v3.9.1) and Enrichment Map (*44*) (v3.3.5) (https://enrichmentmap.readthedocs.io/) with parameters: *P* < 0.001 (node cutoff) and Jaccard Overlap Combined Index (k constant = 0.5) > 0.5 (edge cutoff). Clustering was performed on the graph using MCL Cluster from AutoAnnotate (*62*, *63*) (v1.4.0). Annotations were manually reviewed and edited where appropriate.

### Correlation of host gene expression and microbial load

We used the R function corr.test (with the argument method = “spearman”) to identify correlations between normalized expression for all genes and the number of genera-resolution microbial UMIs. We excluded all cells with zero microbial UMIs to focus on microbial load and avoid identifying genes simply associated with infection. We used the R function corr.test (with the argument method = “spearman”) to compare the correlations for overlapping genes in Pelka2021 and Zhang2021.
