# Supplementary material for: Identification of intracellular bacteria from multiple single-cell RNA-seq platforms using CSI-Microbes
Source: Sci Adv. 2024 Jul 3;10(27):eadj7402. doi: 10.1126/sciadv.adj7402 (PMC11221508; doi:10.1126/sciadv.adj7402)
Supplement: Supplementary file 1 — Figs. S1 to S11 Legends for tables S1 to S6 [file sciadv.adj7402_sm.pdf]

Science Advances
AAAS

Supplementary Materials for

Identification of intracellular bacteria from multiple single-cell RNA-seq platforms using CSI-Microbes

Welles Robinson *et al.*

Corresponding author: Welles Robinson, wir963@gmail.com; Eytan Ruppin, eytan.ruppin@nih.gov

Sci. Adv. **10**, eadj7402 (2024)
DOI: 10.1126/sciadv.adj7402

The PDF file includes:

Figs. S1 to S11
Legends for tables S1 to S6

Other Supplementary Material for this manuscript includes the following:

Tables S1 to S6

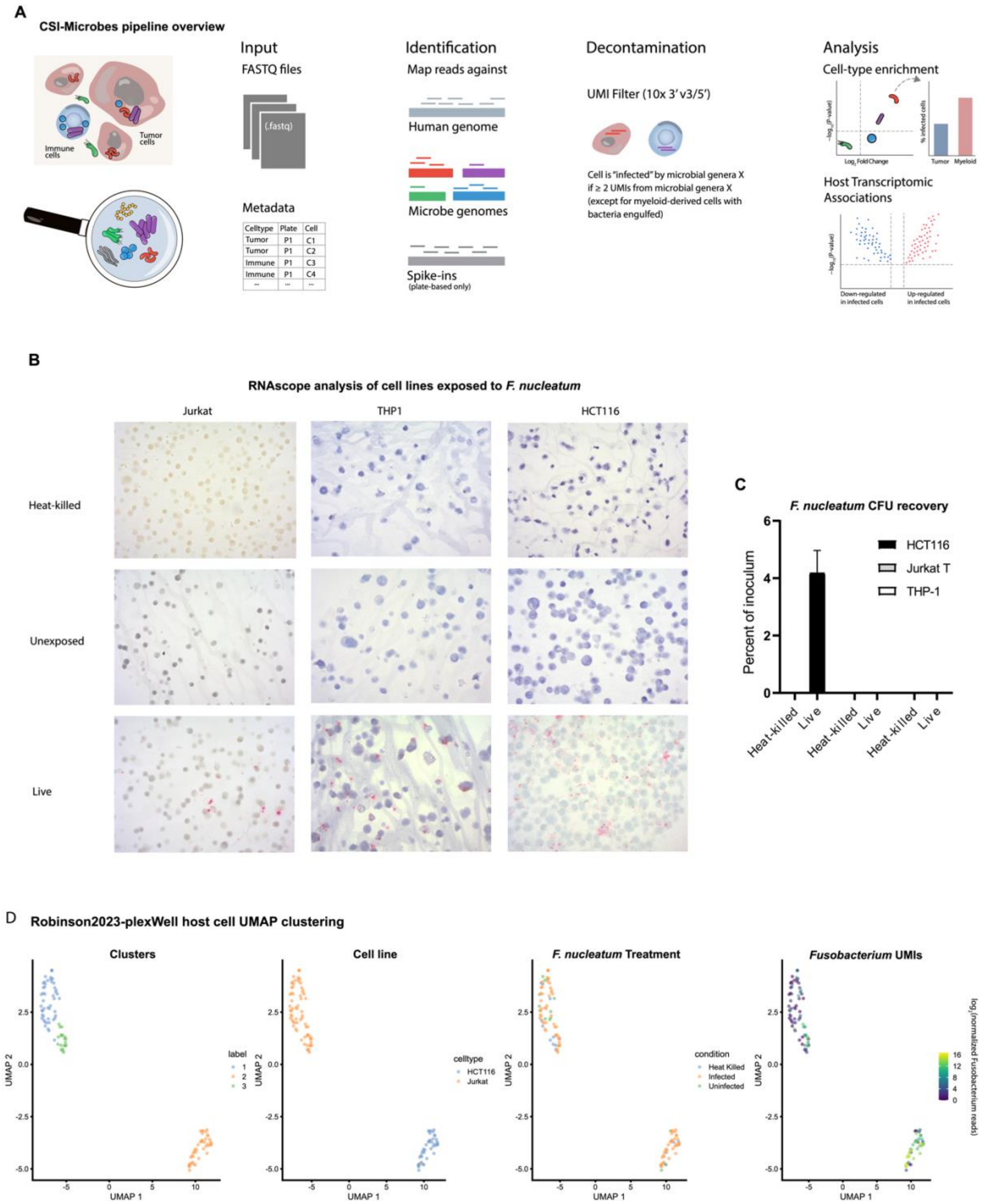

Fig. S1. Overview of CSI-Microbes

(**A**) Overview of the steps in the CSI-Microbes pipeline including input, alignment, decontamination and analysis. (**B**) Representative images of RNAscope analysis using a

Fusobacterium specific RNAscope probe applied to three cell-lines (Jurkat T, THP1 and HCT116) under three infection conditions (unexposed, exposed to heat-killed *F. nucleatum*, exposed to live *F. nucleatum*). (**C**) Bacterial colonies recovered following overnight invasion assay with heat-killed or live *F. nucleatum*. Colony numbers represented as percentage of inoculum at time of infection. Bar graphs represent the mean of three biological replicates, indicated by error bars. (**D**) UMAPs of HCT116 and Jurkat T cells sequenced using plate-based approaches colored by host transcriptomic clustering, cell identity, *F. nucleatum* exposure condition and the number of *Fusobacterium* reads identified per cell.

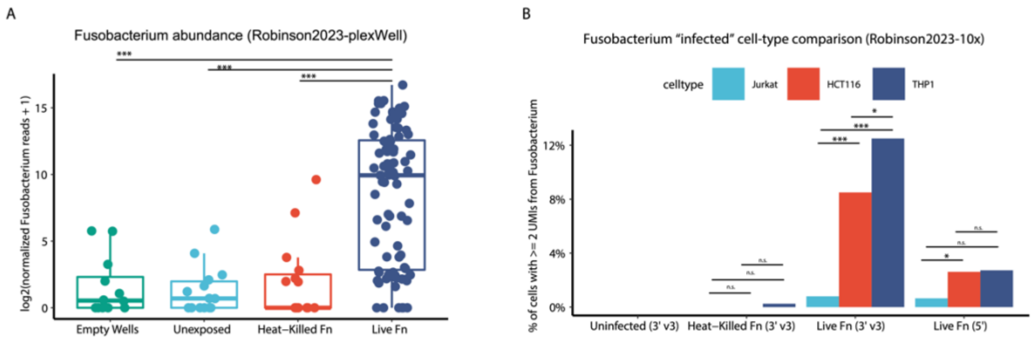

Fig. S2. CSI-Microbes identifies enrichments in cells exposed to live *Fusobacterium*

(**A**) The number of *Fusobacterium* reads per cell across different exposure conditions (regardless of cell type) from Robinson2023-plexWell. (**B**) The percentage of cells infected by *Fusobacterium* per cell type grouped by condition from Robinson2023-10x. * p-value < 0.05; ** p-value < 0.01; *** p-value < 0.001.

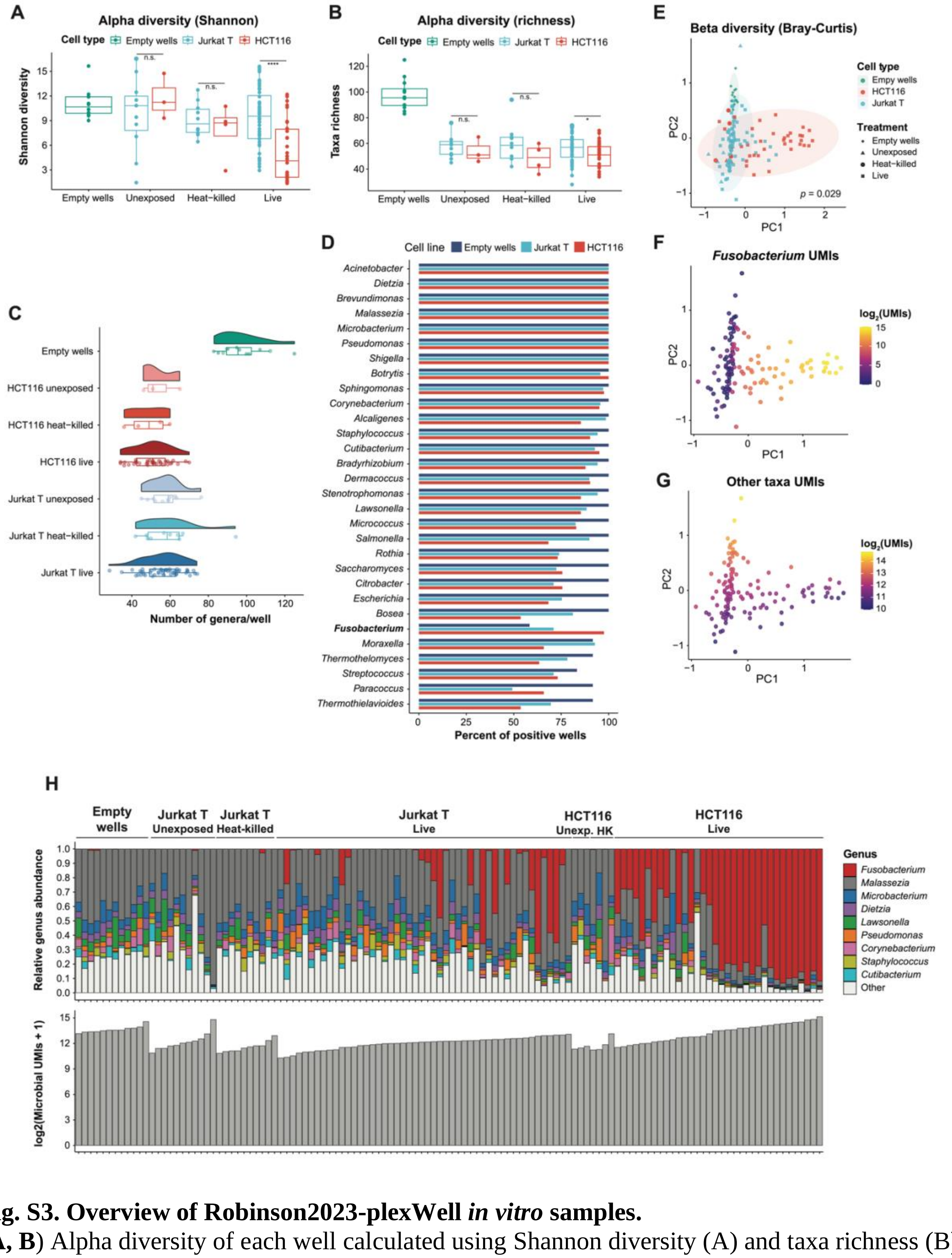

Fig. S3. Overview of Robinson2023-plexWell *in vitro* samples.
(**A, B**) Alpha diversity of each well calculated using Shannon diversity (A) and taxa richness (B). (**C**) Number of unique genera identified per well, stratified by cell line and *F. nucleatum* treatment group. (**D**) Genera prevalence was calculated by the percentage of wells positive for

any given genus by cell line only and then ordered by overall abundance, from most prevalent to least. Top 30 most abundant genera were graphed. (**E**) Beta diversity of each well using the Bray-Curtis method. (**F, G**) The total microbial UMIs for *Fusobacterium* (F) and all other genera (G) were log_2-transformed and placed onto the PC dimensions calculated in (E). (**H**) The relative (top) and absolute (bottom) microbial UMI burden for each well was determined with the top nine most abundant genera shown. n.s. not significant, * $p < 0.05$, **** $p < 0.00001$.

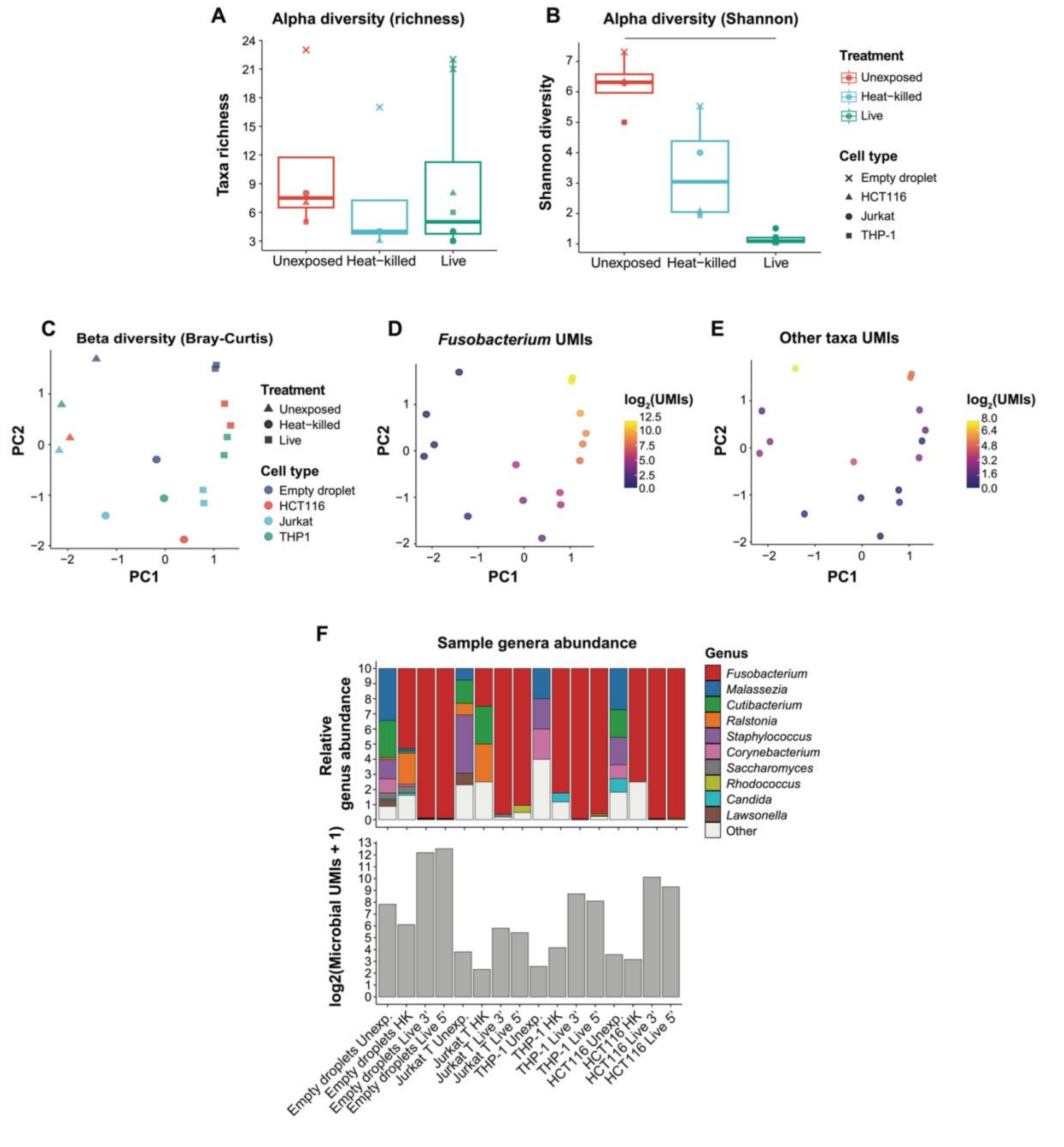

Fig. S4. Overview of Robinson2023-10x *in vitro* samples.
(**A, B**) Alpha diversity of each sample calculated using taxa richness (A) and Shannon (B). (**C**) Beta diversity of each sample using Bray-Curtis method. (**D, E**) The total microbial UMIs for *Fusobacterium* (D) and all other genera (E) were $\log_2$-transformed and placed onto the principal component dimensions calculated in (C). (**F**) The relative (top) and absolute (bottom) microbial UMI burden for each sample was determined with the top nine most abundant genera shown. n.s. not significant, * $p < 0.05$, ** $p < 0.001$.

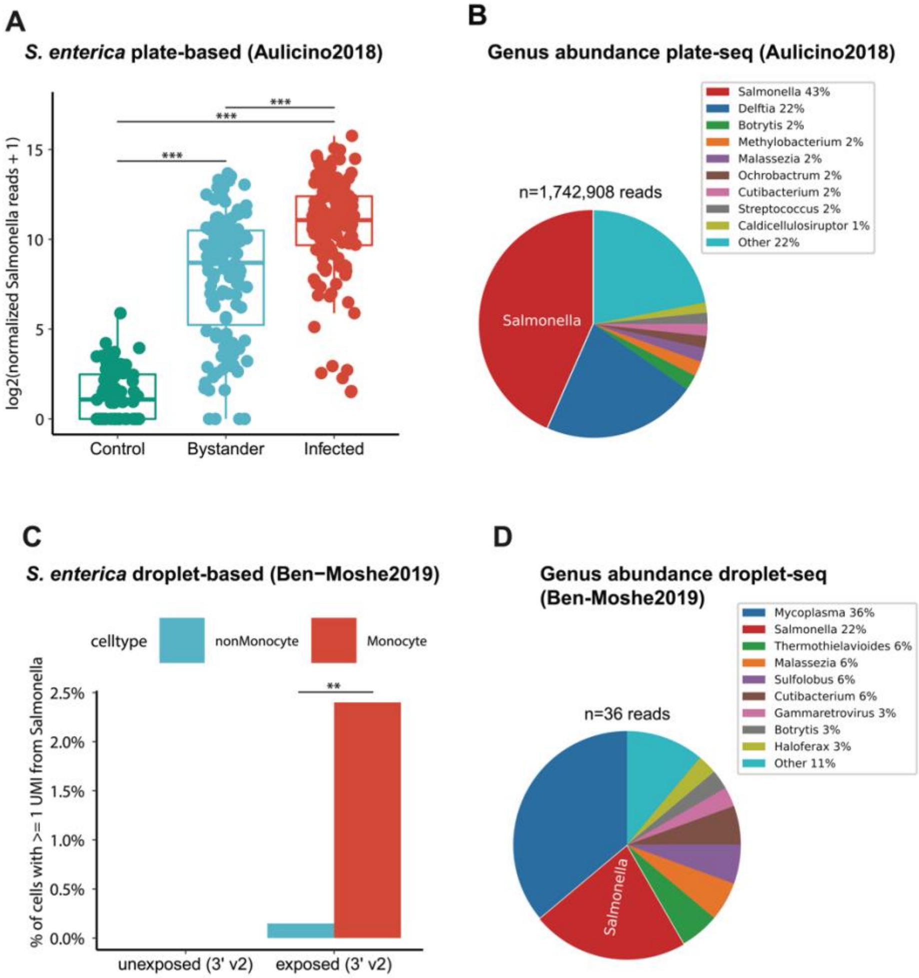

Fig. S5. Other *in vitro* datasets using *Salmonella enterica* infection models

(**A**) The number of reads (spike-in normalized and $\log_2$ transformed) mapping to the genus Salmonella per monocyte-derived dendritic cell (moDC) grouped by exposure condition and sequenced using plate-based scRNA-seq by Aulicino2018. (**B**) The percentage of genera-resolution microbial reads mapped to *Salmonella* and other genera (suspected contaminants) from live-*Se*-exposed cells sequenced using Smart-seq2. (**C**) The percentage of PBMC cells with at least one read from *Salmonella* grouped by cell type and exposure condition sequenced using 10x 3’ v2 by Ben-Moshe2019. (**D**) The percentage of genera-resolution microbial reads mapped to *Salmonella* and other genera (suspected contaminants) from live-*Se*-exposed cells sequenced using 10x 3’ v2. * p-value < 0.05; ** p-value < 0.01; *** p-value < 0.001.

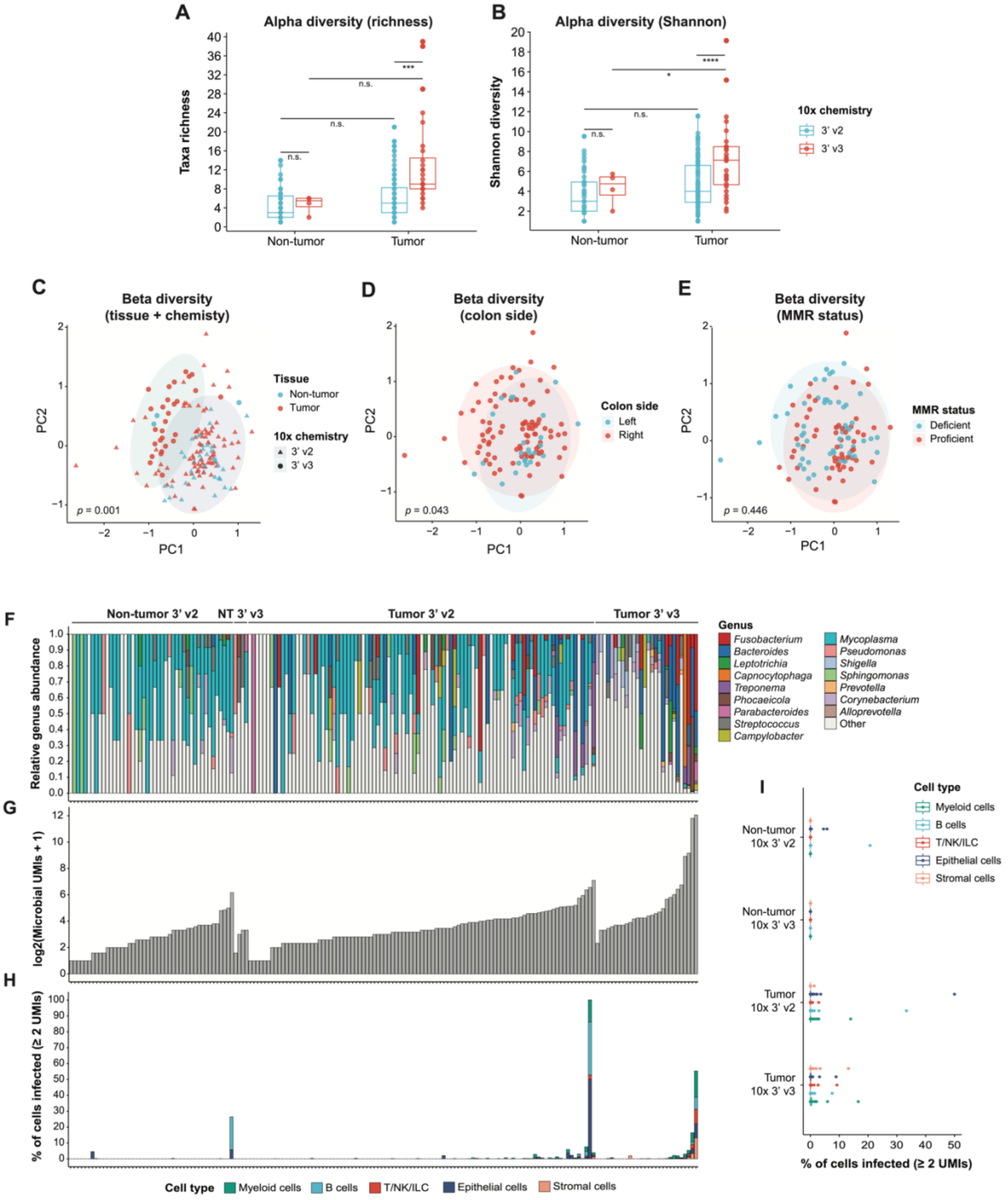

Fig. S6. Overview of Pelka2021 colorectal cancer patient samples.

(A, B) Alpha diversity of each sample calculated using taxa richness (A) and Shannon diversity (B). **(C, D, E)** Beta-diversity of each sample using Bray-Curtis method, by tissue and chemistry (C), colon side for tumor samples only (D), and MMR mutational status for tumor samples only (E). **(F, G)** The relative (F) and absolute (G) microbial UMI burden for each sample. Genera in

(F) were those identified as enriched in myeloid cells in Figure 4B. **(H)** Percentage of cells infected (positive for ≥2 microbial UMIs), separated by cell type as identified by the original authors. The percentage of positive cells was calculated separately for each cell type and then added together. **(I)** Comparison of the percent of infected cells, showing totals for each sample by cell type. n.s. not significant, * $p < 0.05$, *** $p < 0.0001$, **** $p < 0.00001$.

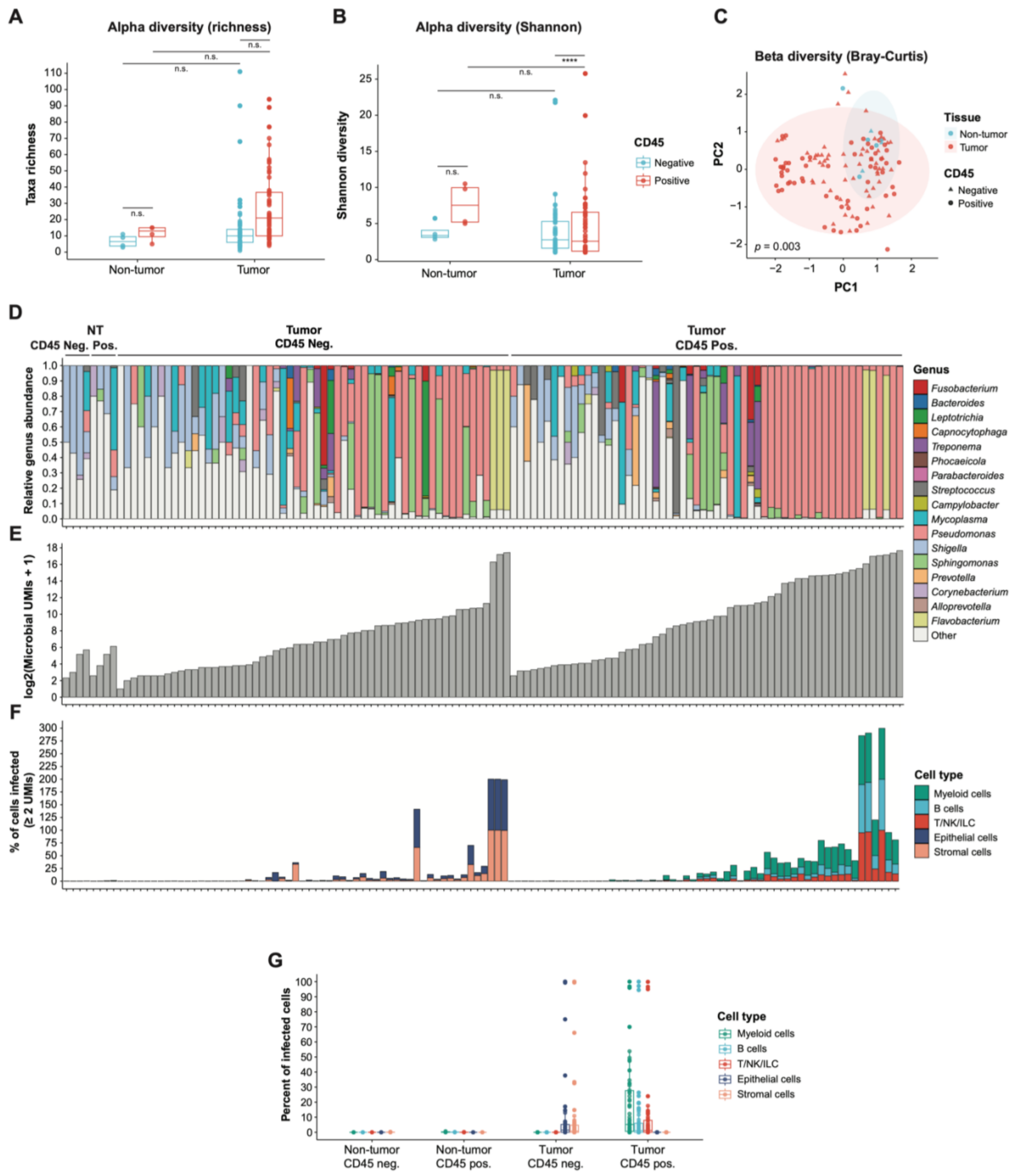

Fig. S7. Overview of Zhang2021 esophageal cancer patient samples.

(A, B) Alpha diversity of each sample calculated using taxa richness (A) and Shannon (B). **(C)** Beta diversity of each sample using Bray-Curtis method, by tissue and CD45 status, as determined by the original authors. **(D, E)** The relative (D) and absolute (E) microbial UMI burden for each sample. Genera in (D) were those identified as enriched in myeloid cells in Figure 4B. **(F)** Percentage of cells infected (positive for ≥2 microbial UMIs), separated by cell

type as identified by the original authors. The percentage of positive cells was calculated separately for each cell type and then added together, giving totals greater than 100%. **(G)** Comparison of the percent of infected cells, showing totals for each sample by cell type. n.s. not significant, **** $p < 0.00001$.

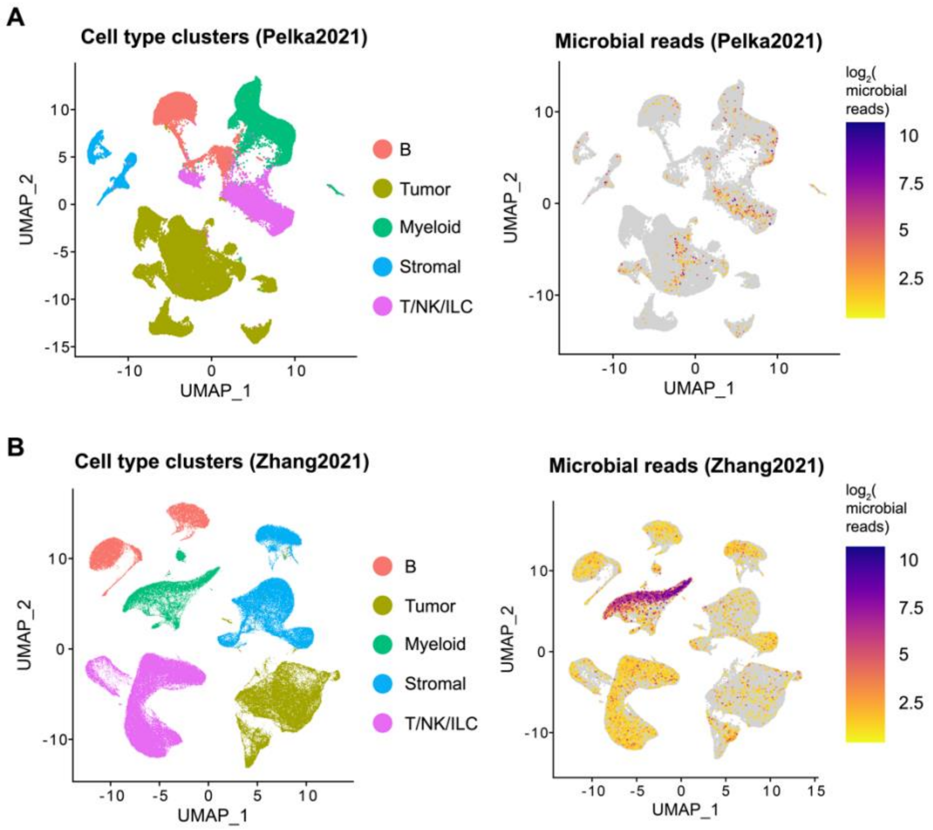

Fig. S8. Extended analysis of patient scRNA-seq datasets (Pelka2021 and Zhang2021)

(A) UMAP analysis of cells analyzed from Pelka2021 colored by the cell type annotations (determined by the original authors) (left) and the number of microbial reads identified by CSI-Microbes. **(B)** UMAP analysis of cells analyzed from Zhang2021 colored by the cell type annotations (determined by the original authors) (left) and the number of microbial reads identified by CSI-Microbes (right).

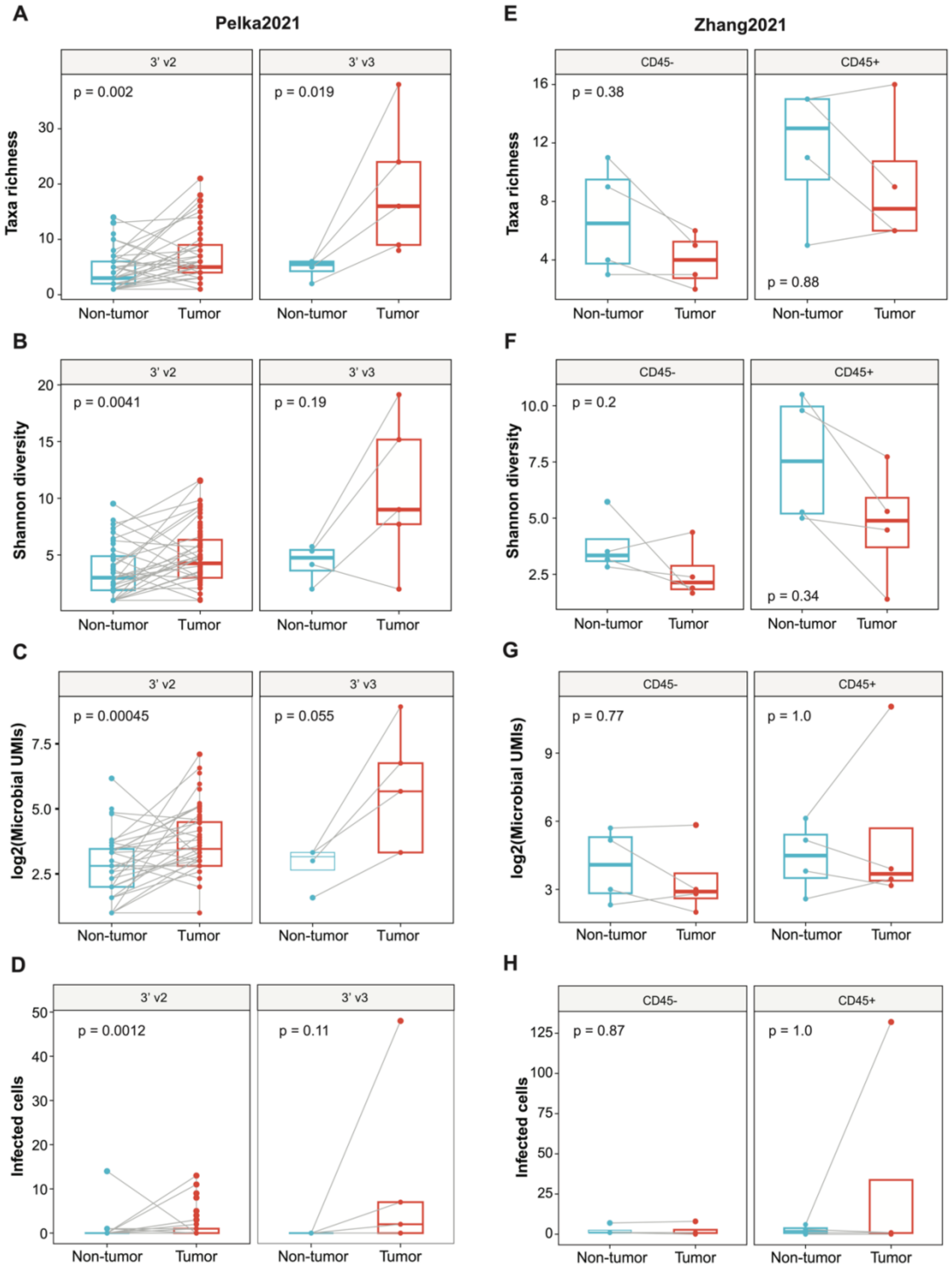

Fig. S9. Paired NT-T samples do not show significant differences in Pelka2021 or Zhang2021

(A, B) Alpha diversity of each sample using taxa richness (A) and Shannon (B) in the Pelka2021 dataset. Non-tumor and tumor samples were paired by patient ID number and then stratified by

10x sequencing chemistry. **(C)** Log_2-transformation of total microbial UMIs in the Pelka2021 dataset and paired by patient ID. **(D)** The number of infected cells per non-tumor or tumor sample in the Pelka2021 dataset. **(E, F, G, H)** Taxa richness (E), Shannon diversity (F), total microbial UMIs (G), and number of infected cells (H) in the Zhang2021 dataset. All paired sample graphs in panels E-H were presented in the same manner as described for Pelka2021, with the exception of partitioning samples according to CD45+ vs. CD45- expression.

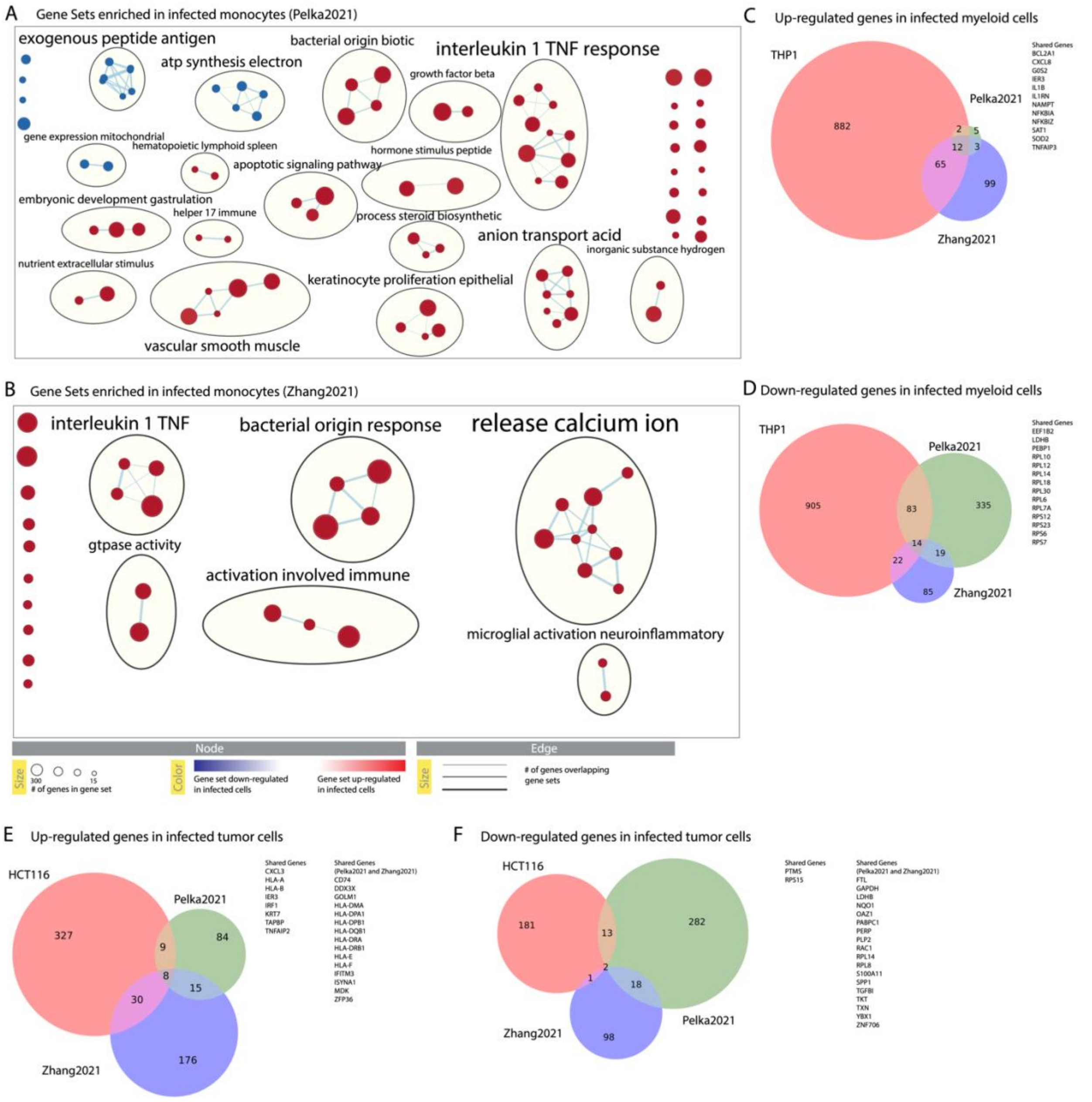

Fig. S10. Extended analysis of host transcriptomic changes associated with bacterial infection.

(A) Annotated clusters of gene ontology (GO) biological processes (BP) gene sets up-regulated (red) or down-regulated (blue) in infected monocytes in Pelka2021. **(B)** Annotated clusters of GO BP gene sets up-regulated (red) or down-regulated (blue) in infected monocytes in Zhang2021. **(C)** The overlap of individual differentially expressed genes (DEGs) up regulated in infected myeloid cells in Pelka2021, infected myeloid cells in Zhang2021 and THP1 cells exposed to live *F. nucleatum* (compared to unexposed THP1 cells). **(D)** The overlap of down-regulated DEGs in infected myeloid cells in Pelka2021, infected myeloid cells in Zhang2021 and THP1 cells exposed to live *F. nucleatum* (compared to unexposed THP1 cells). **(E)** The overlap

of up-regulated DEGs in infected tumor cells in Pelka2021, infected tumor cells in Zhang2021 and HCT116 cells exposed to live *F. nucleatum* (compared to unexposed HCT116 cells). **(F)** The overlap of down-regulated DEGs in infected tumor cells in Pelka2021, infected tumor cells in Zhang2021 and HCT116 cells exposed to live *F. nucleatum* (compared to unexposed HCT116 cells).

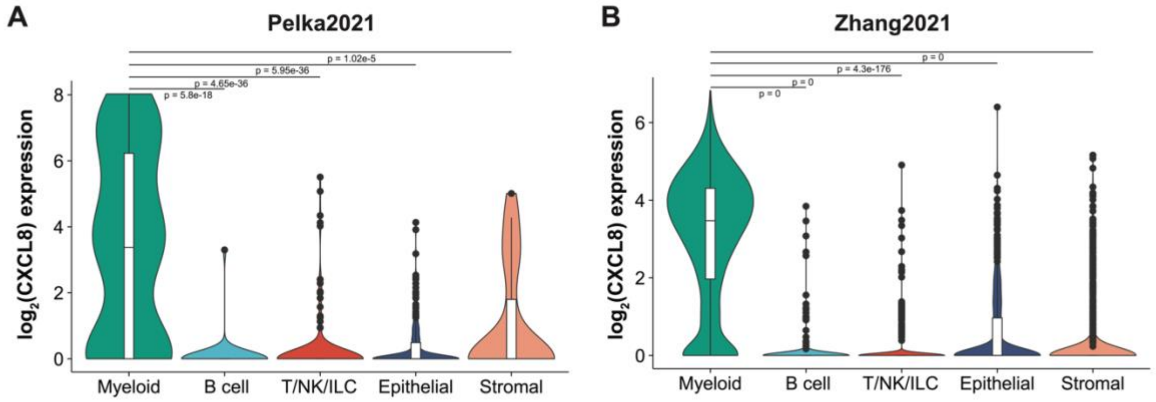

Fig. S11. Infected myeloid cells express higher levels of *CXCL8* compared to other cell types.

(A) Pelka2021 *CXCL8* expression levels were $\log_2$-transformed and cells were filtered for bacterial infection (≥2 microbial UMIs). **(B)** Zhang2021 *CXCL8* expression levels were $\log_2$-transformed and cells were filtered for bacterial infection (≥2 microbial UMIs).

Table S1. Additional data from *in vitro* infection scRNA-seq datasets.

Table S2. Complete genera and celltype enrichment results from Pelka2021 and Zhang2021.

Table S3. Complete co-infection results from Pelka2021 and Zhang2021.

Table S4. Human cell gene expression associations with microbial infection in Pelka2021 and Zhang2021.

Table S5. Gene set enrichment analysis results from Pelka2021 and Zhang2021.

Table S6. Association between human cell gene expression and the number of microbial UMIs ("microbial load") in Pelka2021 and Zhang2021.
